# Supplementary material for: Retromer Controls Planar Polarity Protein Levels and Asymmetric Localization at Intercellular Junctions
Source: Curr Biol. 2019 Feb 4;29(3):484–491.e6. doi: 10.1016/j.cub.2018.12.027 (PMC6370945; doi:10.1016/j.cub.2018.12.027)
Supplement: Document S1. Figures S1–S4 and Table S1 [file mmc1.pdf]

Current Biology, Volume *29*

Supplemental Information

Retromer Controls Planar Polarity Protein Levels and Asymmetric Localization at Intercellular Junctions

Helen Strutt, Paul F. Langton, Neil Pearson, Kirsty J. McMillan, David Strutt, and Peter J. Cullen

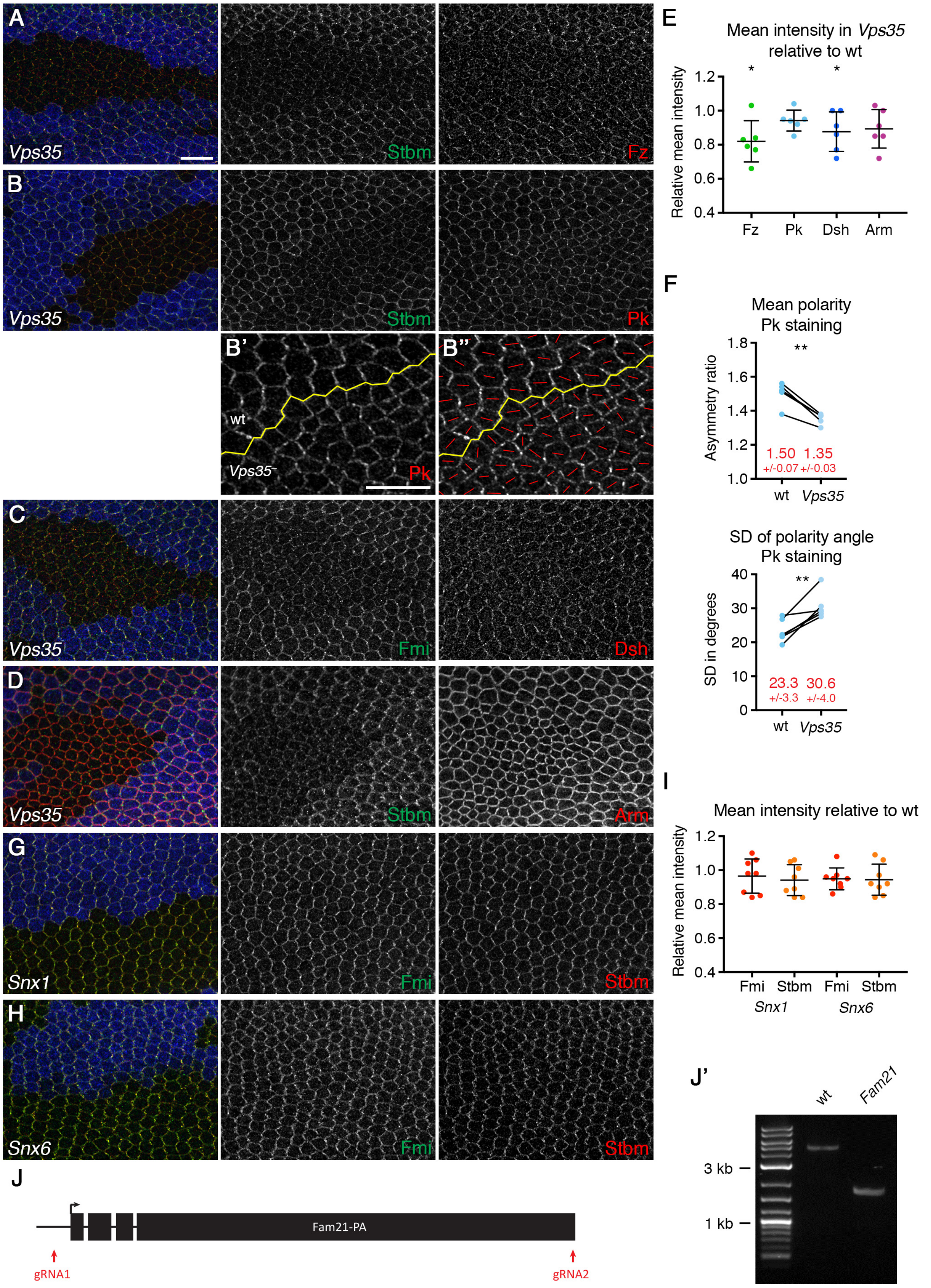

Figure S1. Effects of loss of Vps35, Snx1 and Snx6 on membrane-associated proteins. Related to Figure 1

(A-D, G, H) 28 hr APF pupal wings carrying clones of *Vps35* (A-D), *Snx1* (G) or *Snx6* (H), marked by loss of ß-gal immunolabelling (blue). (A, B, D) Wings immunolabelled for Stbm in green and Fz (A), Pk (B) or Arm (D) in red. (C) Wings immunolabelled for Fmi in green and Dsh in red. (G, H) WIngs immunolabelled for Fmi in

green and Stbm in red. (B') High magnification image of wild-type and mutant regions immunolabelled with Pk and used to quantitate polarity. (B'') Polarity nematic showing the magnitude and angle of polarisation for each cell. Scale bar 10 μm.

(E, I) Quantitation of mean intensity of membrane labelling for the genotypes in A-D, G, H. (E) Wings immunolabelled for Fz (green dots), Pk (pale blue dots), Dsh (dark blue dots) or the non planar polarity protein Armadillo (purple dots). (I) Wings immunolabelled for Fmi (red dots) and Stbm (orange dots). Intensity is shown as a ratio of signal in mutant tissue compared to wild-type in each wing, error bars are SD. One-sample t-tests were used to determine if the ratio differed from 1.0. *$p<0.05$.

(F) Quantitation of polarity in *Vps35* pupal wing clones immunolabelled for Pk, levels of which are not significantly decreased within the clone (see panels B and E). Graphs show mean polarity and variation in polarity angle, in wild-type and *Vps35* mutant tissue. Values from the same wing are linked by black bars, mean and SD are listed. Paired t-tests were used to compare values in the same wing, **$p<0.01$. Like wings immunolabelled for Fmi (Fig. 1E), mean polarity and variation in polarity angle are significantly different in *Vps35* tissue than in wild-type tissue, suggesting that the polarity measurements are not affected by overall levels of protein at the junctions.

(J) Diagram of the *Fam21* locus, showing the exon structure and the position of the gRNAs used for CRISPR/Cas9 gene editing, which results in a replacement of the entire coding sequence of *Fam21*, except the final 36bp, by a pax-mCherry selection cassette. (J') Agarose gel showing PCR products after amplification of genomic DNA from wild-type and *Fam21* homozygous mutant flies. The PCR product in *Fam21* flies is smaller than in wild-type flies, as expected.

See Data S1 for all statistical comparisons.

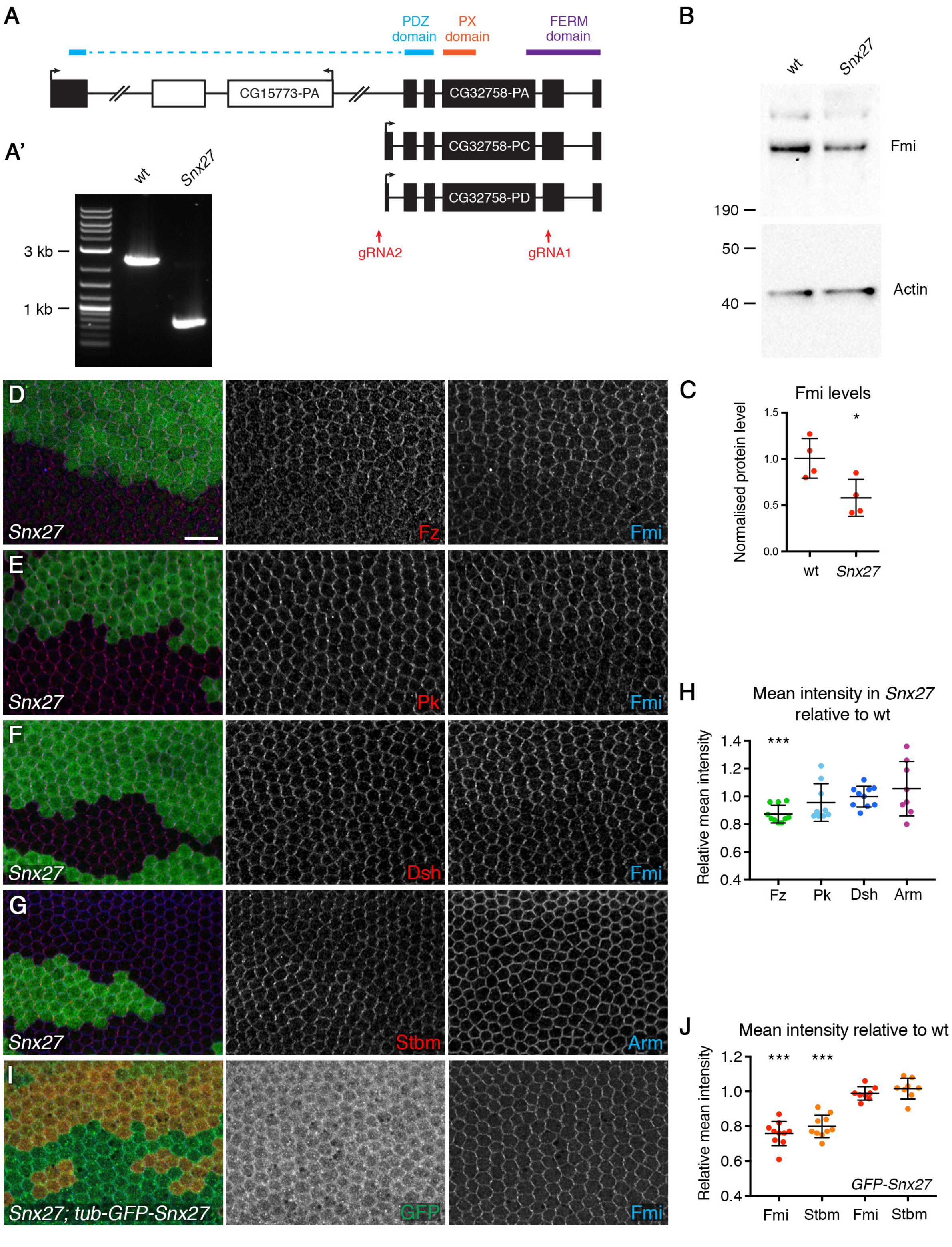

Figure S2. Effect of loss of Snx27 on overall Fmi levels and on other membrane-associated proteins. Related to Figure 3

(A) Diagram of the *Snx27/CG32758* locus showing the 3 predicted isoforms. The sequence encoding the PDZ, PX and FERM domains is indicated above. Another gene (*CG15773*) is present in the first intron of *CG32758-RA*, so the *Snx27* knock-out was made by deleting the C-terminal exons, using two gRNAs targeting the indicated locations. (A') Agarose gel showing PCR products after amplification of genomic DNA from wild-

type and *Snx27* homozygous mutant flies. The PCR product in *Snx27* flies is smaller than in wild-type flies, as expected.

(B-C) Immunoblot (B) and quantitation of protein levels (C) from pupal wing extracts from wild-type and *Snx27* flies, probed for Fmi and Actin. Quantitation was from 4 biological replicates, and samples were compared by unpaired t-test, *$p<0.05$.

(D-G) 28 hr APF pupal wings carrying clones of *Snx27*, marked by loss of GFP (green). (D-F) Wings immunolabelled for Fmi in blue and Fz (A), Pk (B) or Dsh (C) in red. (G) Wings immunolabelled for Stbm in red and the non-planar polarity protein Armadillo in blue. Scale bar 10 μm.

(I) 28 hr APF pupal wing expressing *tub-GFP-Snx27* (GFP immunolabelling in green) and carrying clones of *Snx27*, marked by loss of RFP fluorescence (red). Wings immunolabelled for Fmi in blue.

(H, J) Quantitation of mean intensity of membrane labelling for the genotypes in D-G, I and Figure 3A. (H) Wings immunolabelled for Fz (green dots), Pk (pale blue dots), Dsh (dark blue dots) or Armadillo (purple dots). (J) Wings immunolabelled for Fmi (red dots) or Stbm (orange dots), in the presence or absence of *tub-GFP-Snx27.* Intensity is shown as a ratio of signal in *Snx27* mutant compared to wild-type in each wing, error bars are SD. One-sample t-tests were used to determine if the ratio differed from 1.0. ***$p<0.001$.

See Data S1 for all statistical comparisons.

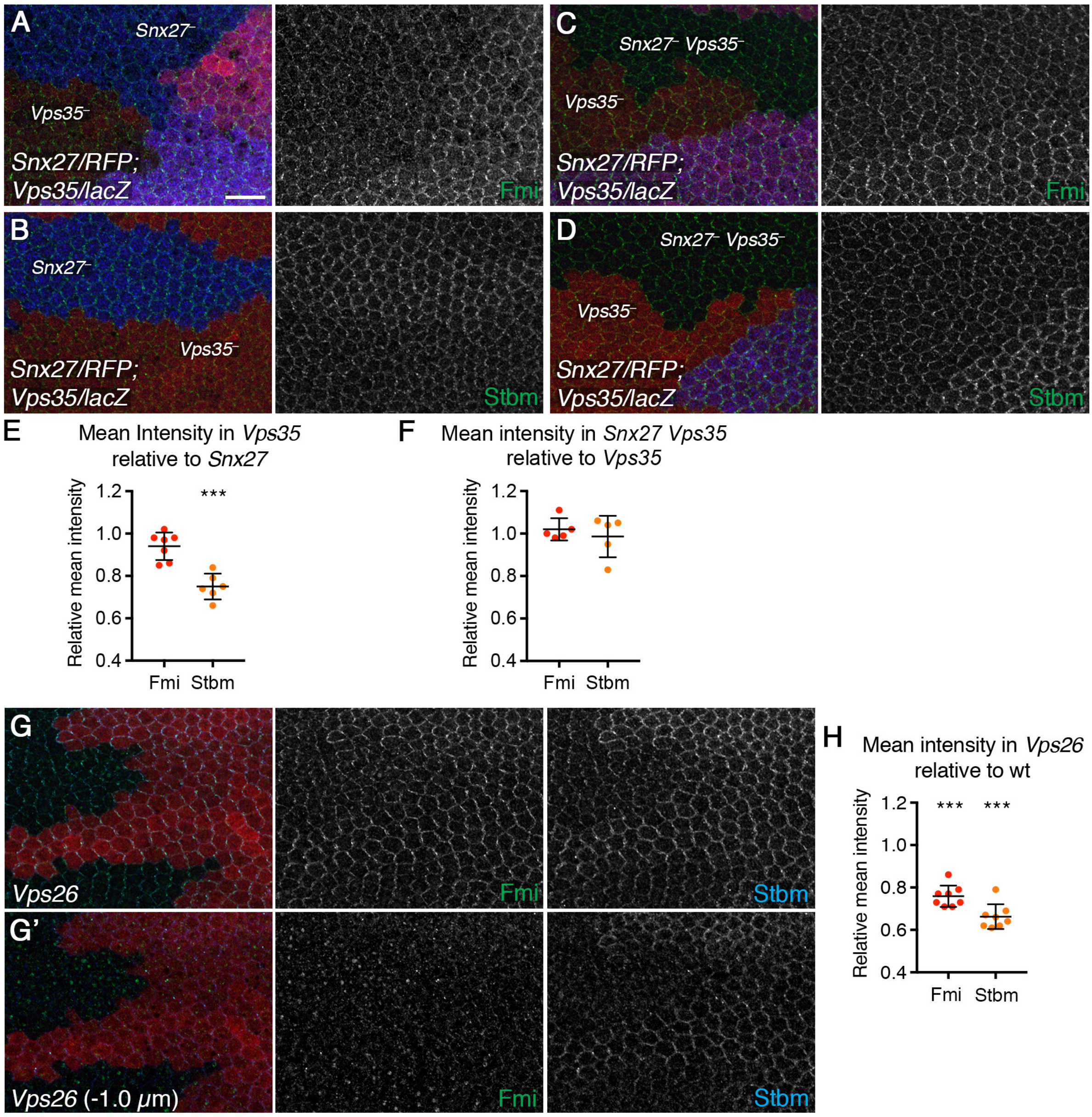

Figure S3. Stbm levels are regulated by retromer independently of Snx27. Related to Figure 3

(A, B, C, D) 28 hr APF pupal wings carrying clones of *Snx27* marked by loss of RFP fluorescence (red), and clones of *Vps35^{MH20}* marked by loss of ß-gal immunolabelling (blue). Wings immunolabelled for Fmi (A, C) or Stbm (B, D) in green. (A, B) *Snx27* clones adjacent to *Vps35* clones. Stbm levels are reduced in *Vps35* clones compared to *Snx27* clones, while Fmi levels are similar. (C, D) Clones of *Vps35* (loss of blue labelling) next to tissue mutant for both *Snx27* and *Vps35* (loss of red and blue labelling). Levels of Fmi and Stbm are similar in both regions. Scale bar 10 µm.

(E, F) Quantitation of mean intensity of Fmi (red dots) or Stbm (orange dots) immunolabelling at membranes for the immunolabellings shown in A, B, C and D. Intensity is shown as a ratio of signal in *Vps35* mutant compared to *Snx27* mutant in each wing (E), or as ratio of signal in *Snx27 Vps35* double mutant compares to *Vps35* single mutant (F). Error bars are SD, one-sample t-tests were used to determine if the ratio differed from 1.0, ***$p<0.001$.

(G) 28 hr APF pupal wings carrying clones of *Vps26*, marked by loss of RFP fluorescence (red). WIngs immunolabelled for Fmi in green and Stbm in blue. (G) shows immunolabelling in the junctional region and (G') shows immunolabelling more basally. Intracellular puncta containing Fmi, but not Stbm, are visible in *Vps26* mutant tissue. Such large intracellular puncta are never seen in *Snx27* mutant tissue, possibly because Fmi never engages with the recycling machinery in this situation and is rapidly degraded in the lysosome. Intracellular

puncta are also rarely seen in *Vps35* mutant tissue. We hypothesise that in *Vps26* mutant tissue, Fmi binds to its cargo adaptor Snx27 and is retained in the endosomal system.

(H) Quantitation of mean intensity of Fmi (red dots) or Stbm (orange dots) membrane labelling in pupal wing clones of *Vps26*. Intensity is shown as a ratio of signal in *Vps26* mutant compared to wild-type in each wing. Error bars are SD, one-sample t-tests were used to determine if the ratio differed from 1.0, ***$p<0.001$.

See Data S1 for all statistical comparisons.

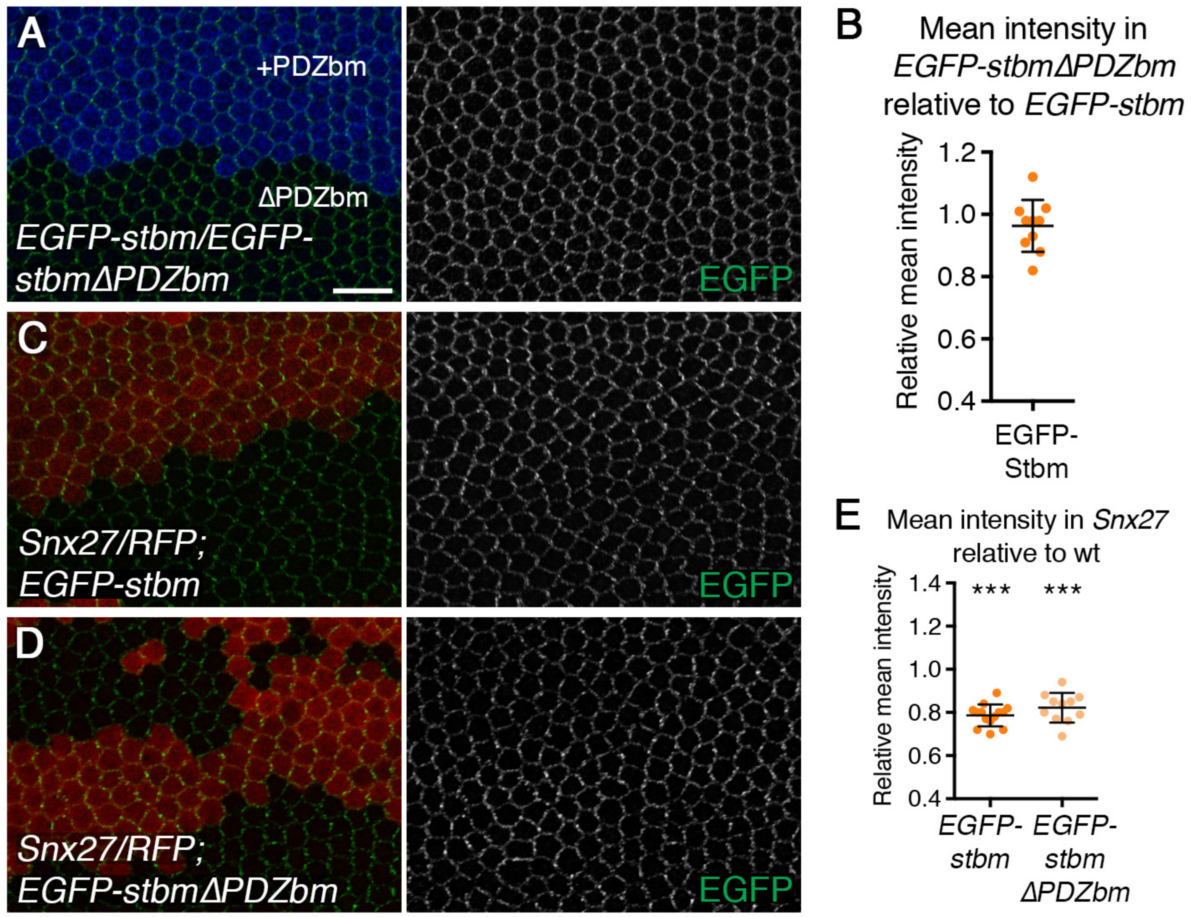

Figure S4. Stbm lacking its PDZ binding motif is sensitive to loss of Snx27. Related to Figure 3

(A) 28 hr APF pupal wings with twin clones of *P[acman]-EGFP-stbm*, marked by ß-gal immunolabelling in blue, next to *P[acman]-EGFP-stbm*Δ*PDZ binding motif* (ΔPDZbm), in a *stbm*[6] mutant background. Images immunolabelled for EGFP in green. Scale bar 10 µm.

(B) Quantitation of mean intensity of membrane labelling of EGFP-Stbm (A). Intensity is shown as a ratio of signal in ΔPDZbm compared to full-length protein in each wing, error bars are SD. One-sample t-tests were used to determine if the ratio differed from 1.0, no significant differences were seen.

(C, D) 28 hr APF pupal wings expressing *P[acman]-EGFP-stbm* (C) or *P[acman]-EGFP-stbm* Δ*PDZ binding motif (*Δ*PDZbm)* (D), in a *stbm*[6] mutant background. Clones of *Snx27* are marked by loss of RFP (red) and EGFP fluorescence is in green. Levels of both EGFP-Stbm and EGFP-StbmΔPDZbm decrease in *Snx27* clones.

(E) Quantitation of mean intensity of EGFP-Stbm at membranes for the genotypes in C and D. Intensity is shown as a ratio of EGFP signal in *Snx27* mutant compared to wild-type in each wing. Error bars are SD. One-sample t-tests were used to determine if the ratio differed from 1.0, ***$p<0.001$.

See Data S1 for all statistical comparisons.

	**wild-type**	***EGFP-fmi***	**Significance**
**Plateau**	**0.60**<br>0.50 - 1.22	**0.30**<br>0.28 - 0.37	**0.006**
**Fast half-life**	**9.2 s**<br>2.4 - 21.8 s	**2.7 s**<br>0.0 - 11.2 s	**0.23**
**Slow half-life**	**313 s**<br>204 - 1029 s	**144 s**<br>107 - 292 s	**0.07**
**Percent Fast recovery**	**15.2**<br>11.9 - 18.5	**24.1**<br>18.9 - 32.2	**different**<br>(model for Percent Fast the same cannot be fitted}

Table S1. Summary of FRAP data. Related to Figure 4

Recovery was fitted to a two-phase exponential curve. Plateau values, fast and slow half lives and the percentage recovery due to the fast phase are shown, together with 95% confidence intervals. Curves were compared using an extra-sum-of squares F test. Note that the curves do not reach a plateau in the time course of the experiment, so the plateaux and half lives are extrapolated, leading to wide 95% confidence intervals. Related to Figure 4I.
