# Supplementary material for: Retromer Controls Planar Polarity Protein Levels and Asymmetric Localization at Intercellular Junctions
Source: Curr Biol. 2019 Feb 4;29(3):484–491.e6. doi: 10.1016/j.cub.2018.12.027 (PMC6370945; doi:10.1016/j.cub.2018.12.027)
Supplement: Document S2. Article plus Supplemental Information [file mmc4.pdf]

Report

Current Biology

Retromer Controls Planar Polarity Protein Levels and Asymmetric Localization at Intercellular Junctions

Highlights

- Retromer regulates levels and asymmetry of Flamingo and Strabismus in the pupal wing
- Retromer regulates Flamingo and Strabismus levels independently of the Wash complex
- The cargo adaptor Snx27 interacts directly with the PDZ-binding motif of Flamingo
- Loss of Snx27 disrupts Flamingo trafficking

Authors

Helen Strutt, Paul F. Langton, Neil Pearson, Kirsty J. McMillan, David Strutt, Peter J. Cullen

Correspondence

d.strutt@sheffield.ac.uk (D.S.),
pete.cullen@bristol.ac.uk (P.J.C.)

In Brief

Planar cell polarity—the organization of cells within a two-dimensional plane—is an important feature in tissue organization. Strutt et al. show that, in the *Drosophila* wing, endosomal recycling through retromer and its cargo adaptor Snx27 mediates the cell-surface localization of two key planar polarity proteins, Flamingo and Strabismus.

Strutt et al., 2019, Current Biology *29*, 484–491
February 4, 2019 Crown Copyright © 2019 Published by Elsevier Ltd.
https://doi.org/10.1016/j.cub.2018.12.027

Current Biology
Report

Retromer Controls Planar Polarity Protein Levels and Asymmetric Localization at Intercellular Junctions

Helen Strutt,[1,3] Paul F. Langton,[2,3] Neil Pearson,[2,3] Kirsty J. McMillan,[2] David Strutt,[1,4,*] and Peter J. Cullen[2,4,5,*]
[1]Department of Biomedical Science, Firth Court, University of Sheffield, Sheffield S10 2TN, UK
[2]School of Biochemistry, Biomedical Sciences Building, University of Bristol, Bristol BS8 1TD, UK
[3]These authors contributed equally
[4]Senior author
[5]Lead Contact
*Correspondence: d.strutt@sheffield.ac.uk (D.S.), pete.cullen@bristol.ac.uk (P.J.C.)
https://doi.org/10.1016/j.cub.2018.12.027

SUMMARY

The coordinated polarization of cells in the plane of a tissue, termed planar polarity, is a characteristic feature of epithelial tissues [1]. In the fly wing, trichome positioning is dependent on the core planar polarity proteins adopting asymmetric subcellular localizations at apical junctions, where they form intercellular complexes that link neighboring cells [1–3]. Specifically, the seven-pass transmembrane protein Frizzled and the cytoplasmic proteins Dishevelled and Diego localize to distal cell ends, the four-pass transmembrane protein Strabismus and the cytoplasmic protein Prickle localize proximally, and the seven-pass transmembrane spanning atypical cadherin Flamingo localizes both proximally and distally. To establish asymmetry, these core proteins are sorted from an initially uniform distribution; however, the mechanisms underlying this polarized trafficking remain poorly understood. Here, we describe the identification of retromer, a master controller of endosomal recycling [4–6], as a key component regulating core planar polarity protein localization in *Drosophila*. Through generation of mutants, we verify that loss of the retromer-associated Snx27 cargo adaptor, but notably not components of the Wash complex, reduces junctional levels of the core proteins Flamingo and Strabismus in the developing wing. We establish that Snx27 directly associates with Flamingo via its C-terminal PDZ binding motif, and we show that Snx27 is essential for normal Flamingo trafficking. We conclude that Wash-independent retromer function and the Snx27 cargo adaptor are important components in the endosomal recycling of Flamingo and Strabismus back to the plasma membrane and thus contribute to the establishment and maintenance of planar polarization.

RESULTS AND DISCUSSION

The endosomal sorting of internalized cargo for recycling to the cell surface is a highly regulated process [7]. A master conductor for the recycling of numerous cargoes is retromer, a stable heterotrimer of VPS29, VPS35, and VPS26 [4, 5]. To examine the role of retromer in the establishment and maintenance of planar polarization (Figures 1A and 1B), we performed genetic analysis in *Drosophila.* As null mutants of the retromer subunit Vps35 are lethal during late larval or early pupal stages [8, 9], we generated *Vps35*-null mutant clones in the pupal wing. This revealed that junctional levels of the transmembrane core proteins Flamingo (Fmi), Strabismus (Stbm), and Frizzled (Fz) were decreased within *Vps35*-null clones (Figures 1C, 1D, and S1A–S1E). Levels of the cytoplasmic core protein Dishevelled (Dsh) were also slightly reduced, and levels of Prickle (Pk) and other junctional proteins, such as Armadillo, were unaffected (Figures S1B–S1E). In contrast, loss of the retromer-linked SNX-BAR proteins, *Snx1* or *Snx6*, or the core component of the retriever complex *C16orf62* (data not shown) did not affect levels of Fmi or Stbm (Figures S1G–S1I). Notably, both core protein asymmetry and polarity coordination between cells (Figures 1E and S1F) were reduced in *Vps35* mutant tissue, accompanied by a delay in trichome initiation (Figure 1F). By revealing a role for retromer in regulating the cell surface levels and asymmetry of core planar polarity proteins in the pupal wing, these data extend the role of retromer in specifying polarity through recycling of Crumbs [10, 11] and the Scribble polarity module [12].

In mammalian cells, retromer function is coupled to the actin-polymerizing Wiskott-Aldrich syndrome and SCAR homolog (WASH) complex, a pentameric assembly of WASH (WASHC1), FAM21 (WASHC2), CCDC53 (WASHC3), SWIP (WASHC4), and Strumpellin (WASHC5) [13, 14]. Within this complex, WASH stimulates the ARP2/3 complex to drive polymerization of branched actin networks that aid the organization of endosomal retrieval sub-domains [7]. We used CRISPR/Cas9 editing to generate a *Drosophila Fam21*-null mutant (see Figure S1J; STAR Methods). Homozygous *Fam21*-null flies were viable and fertile. In *Fam21*-null mutant clones in the pupal wing, we observed no effect on the levels of Fmi and Stbm (Figures 1D and 1G) nor was any effect observed in *wash*-null mutant clones (Figures 1D and 1H) [15]. Therefore, in contrast to *Drosophila* trachea development,

 Crown Copyright © 2019 Published by Elsevier Ltd.
This is an open access article under the CC BY license (http://creativecommons.org/licenses/by/4.0/).

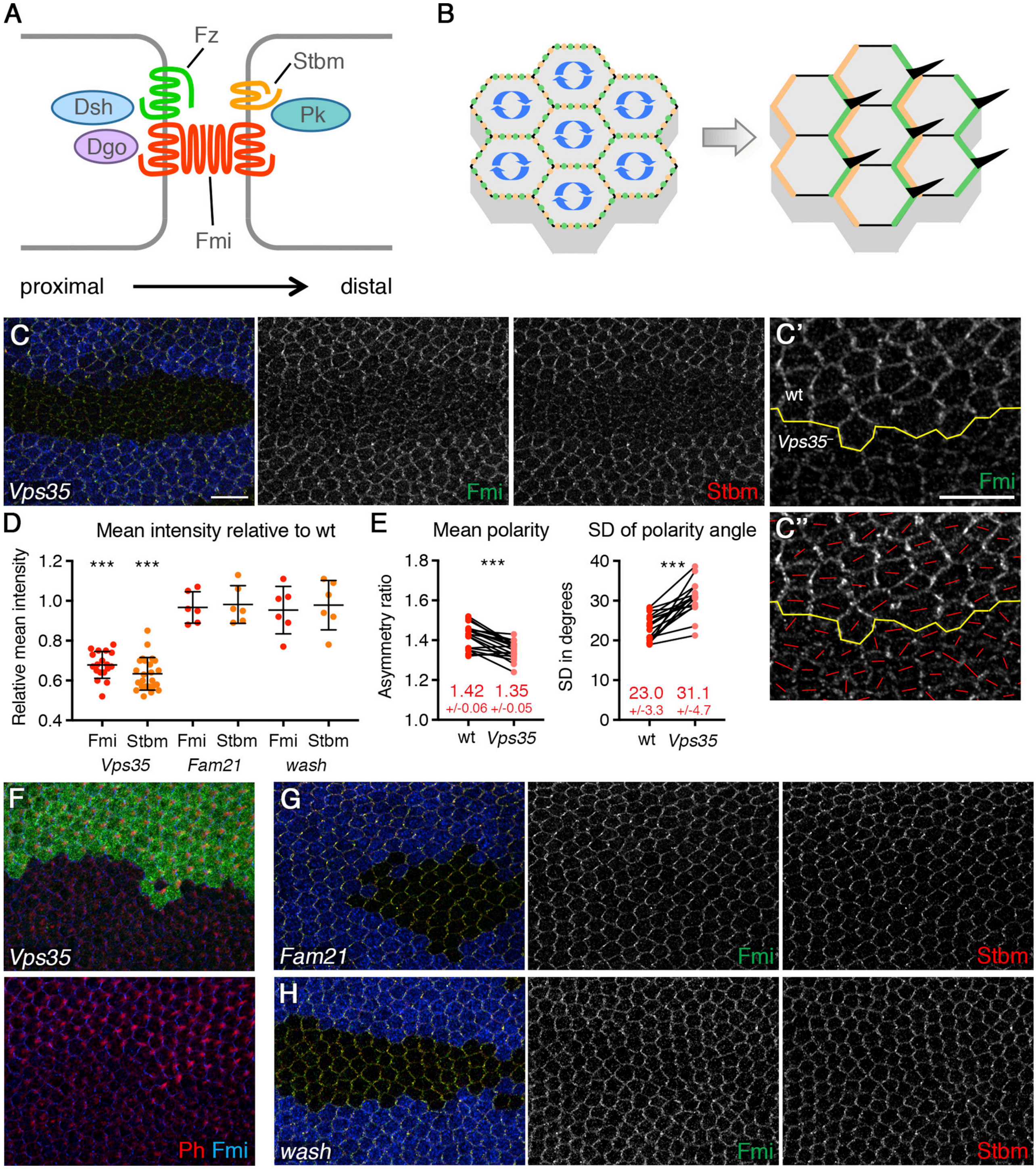

Figure 1. Vps35 Regulates Levels of Fmi and Stbm at Apical Junctions Independently of the Wash Complex

(A) Diagram illustrating asymmetric localization of the core planar polarity proteins in the pupal wing. Two cells are shown, with Fmi, Fz, Dsh, and Dgo localizing on distal cell edges. This forms an intercellular complex with Fmi, Stbm, and Pk on proximal edges of the neighboring cell.

(B) During polarization, complexes sort from a uniform distribution (left), and all the complexes become oriented in the same direction (right). This specifies positioning of trichomes (black in right diagram) to distal cell edges.

(C, F, G, and H) 28-hr after puparium formation (APF) (C, G, and H) or 32-hr APF (F) pupal wings carrying clones of *Vps35* (C and F), *Fam21* (G), or *wash* (H), marked by loss of β-gal staining (blue in C, G, and H and green in F). Wings are immunolabeled for Fmi in green and Stbm in red (C, G, and H) or Fmi in blue and phalloidin in red (F). The reduced phalloidin staining in *Vps35* mutant tissue (F) indicates a delay in trichome initiation. In older wings, phalloidin-stained trichomes are visible in *Vps35* clones (not shown). Scale bar 10 μm.

(legend continued on next page)

where retromer and Wash work together [16, 17], the retromer-mediated asymmetry of Fmi and Stbm in the pupal wing occurs independently of the Wash complex.

A global proteomic analysis, performed *in vitro* and in non-polarized cultured cells, identified an association between CELSR1 and VANGL1/VANGL2, mammalian equivalents of *Drosophila* Fmi and Stbm, respectively, with the retromer cargo adaptor sorting nexin 27 (SNX27) [18]. SNX27 directly associates with the VPS26 subunit of retromer through a mechanism that is conserved in *Drosophila* [19] and promotes endosomal recycling to the plasma membrane. SNX27 contains an amino-terminal PDZ domain that binds to cargoes that contain a C-terminal PDZ binding motif [18, 20]. *Drosophila* Fmi and Stbm both contain PDZ binding motifs (Figure 2A), and they were both able to associate with human SNX27 and *Drosophila* Snx27 in a PDZ-binding-motif-dependent manner (Figures 2B–2E).

To establish the role of *Drosophila* Snx27 in retromer-mediated sorting of Fmi and Stbm, we generated a *Snx27* loss-of-function mutant (see Figure S2A; STAR Methods). *Snx27*-null flies are homozygous viable and fertile. As in *Vps35* mutant clones, junctional levels of both Fmi and Stbm were reduced in *Snx27* pupal wing clones (Figures 3A and 3B). Western blotting revealed that total levels of Fmi were reduced in *Snx27* mutant pupal wings (Figures S2B and S2C), consistent with increased Fmi degradation in the absence of Snx27-retromer-mediated recycling. Levels of Fz at apical junctions were also slightly reduced, and levels of other junctional proteins (Pk, Dsh, and Armadillo) were unaffected (Figures S2D–S2H). Despite the reduced protein levels, core protein asymmetry and trichome initiation and polarity were normal (Figures 3C and 3D), suggesting that sufficient protein reaches the junctions for cells to polarize correctly. The reduced junctional levels of Fmi and Stbm were fully rescued by expression of a rescue transgene *tub-GFP-Snx27* (Figures S2I and S2J), confirming that these effects are due to loss of *Snx27*.

Interestingly, Stbm levels appeared more greatly reduced in *Vps35* clones than in *Snx27* clones, but Fmi levels were similar in each (compare Figures 1D and 3B). This was confirmed by making overlapping clones of *Vps35* and *Snx27* in the same wings (Figures S3A, S3B, and S3E). This suggests that, although retromer acts together with Snx27 to regulate Fmi and Stbm levels, Vps35 also acts on Stbm independently of Snx27. Furthermore, this might explain why reduced asymmetry is seen in *Vps35* mutants, but not in *Snx27* mutants. This Snx27-independent effect of Vps35 is likely to be a retromer-dependent function, as Stbm levels are also decreased more than Fmi levels in *Vps26* mutant tissue (Figures S3G and S3H). Interestingly, Fmi also accumulated in intracellular puncta in *Vps26* mutant tissue (Figure S3G'). Simultaneous loss of *Snx27* and *Vps35* did not cause a further decrease in levels of Fmi and Stbm, over loss of *Vps35* alone (Figures S3C, S3D, and S3F). This suggests that Snx27 acts only through retromer in regulating Fmi and Stbm recycling.

As Snx27 interacts with the PDZ binding motifs of Fmi and Stbm and is required for their retromer-mediated recycling, we examined whether the PDZ binding motifs of these proteins were necessary for their correct junctional levels. First, we generated flies carrying *P[acman]* rescue constructs, in which either full-length Stbm or Stbm lacking the PDZ binding motif was tagged with EGFP at the N terminus. We then generated pupal wings in which clones of EGFP-Stbm were juxtaposed to clones of EGFP-StbmΔPDZ binding motif, in a *stbm* mutant background. No difference in junctional levels of Stbm was seen (Figures S4A and S4B), suggesting that the PDZ binding motif of Stbm is not required for maintenance of Stbm junctional levels and by extension that Snx27 cannot be regulating Stbm levels via a direct interaction between the PDZ binding motif and the PDZ domain. To confirm this, clones of *Snx27* were induced in tissue expressing either EGFP-Stbm or EGFP-StbmΔPDZ binding motif. In both cases, junctional levels were reduced (Figures S4C–S4E), suggesting that the effect of Snx27 on Stbm is independent of the PDZ binding motif.

Next, we generated flies in which the Fmi was tagged with EGFP in its extracellular domain, expressed under the *armadillo* promoter. Clones of EGFP-Fmi adjacent to clones of EGFP-FmiΔPDZ binding motif were then created, in a *fmi* mutant background. Deletion of the PDZ binding motif caused a reduction in junctional levels of Fmi (Figures 3E and 3F). Lower levels of Stbm were also observed at junctions in EGFP-FmiΔPDZ binding motif clones (Figures 3E and 3F), consistent with the previous observation that Fmi is required for normal Stbm levels at the plasma membrane [21] and suggesting that Stbm levels decrease in *Snx27* clones as a consequence of a decrease in Fmi levels. Deletion of the PDZ binding motif of Fmi, although lowering junctional levels of Fmi, did not reduce overall asymmetry (Figure 3G), similar to what is observed in *Snx27* mutants and also in flies with reduced *fmi* gene dosage [22].

The similar phenotypes observed upon loss of *Snx27* and loss of the Fmi PDZ binding motif are consistent with Fmi interacting directly with Snx27 via the PDZ domain of Snx27. Confirming this, we found that EGFP-FmiΔPDZ binding motif was insensitive to loss of Snx27: although junctional levels of EGFP-Fmi were reduced in Snx27 clones (Figures 3H and 3I), levels of EGFP-FmiΔPDZ binding motif did not decrease any further (Figures 3H and 3J).

These data are consistent with the reduced junctional levels of Fmi being caused by reduced recycling, but it is also possible that there is less delivery of newly synthesized Fmi. To distinguish these possibilities, we analyzed Fmi protein dynamics. First, in an antibody internalization experiment, live prepupal

(C') High magnification image of wild-type and mutant regions immunolabeled with Fmi and used to quantitate polarity.
(C'') Polarity nematic showing the magnitude and angle of polarization for each cell.
(D) Quantitation of mean intensity of Fmi (red dots) or Stbm (orange dots) membrane labeling in pupal wing clones. Intensity is shown as a ratio of signal in mutant compared to wild-type in each wing; error bars are SD. One-sample t tests were used to determine whether the ratio differed from 1.0.
(E) Mean polarity and variation in polarity angle of wings immunolabeled for Fmi in wild-type and *Vps35* mutant tissue (see C' and C''). Values from the same wing are linked by black bars; mean and SD are listed. Paired t tests were used to compare values in the same wing. ***$p < 0.001$.
See also Figure S1 and Data S1 for all statistical comparisons.

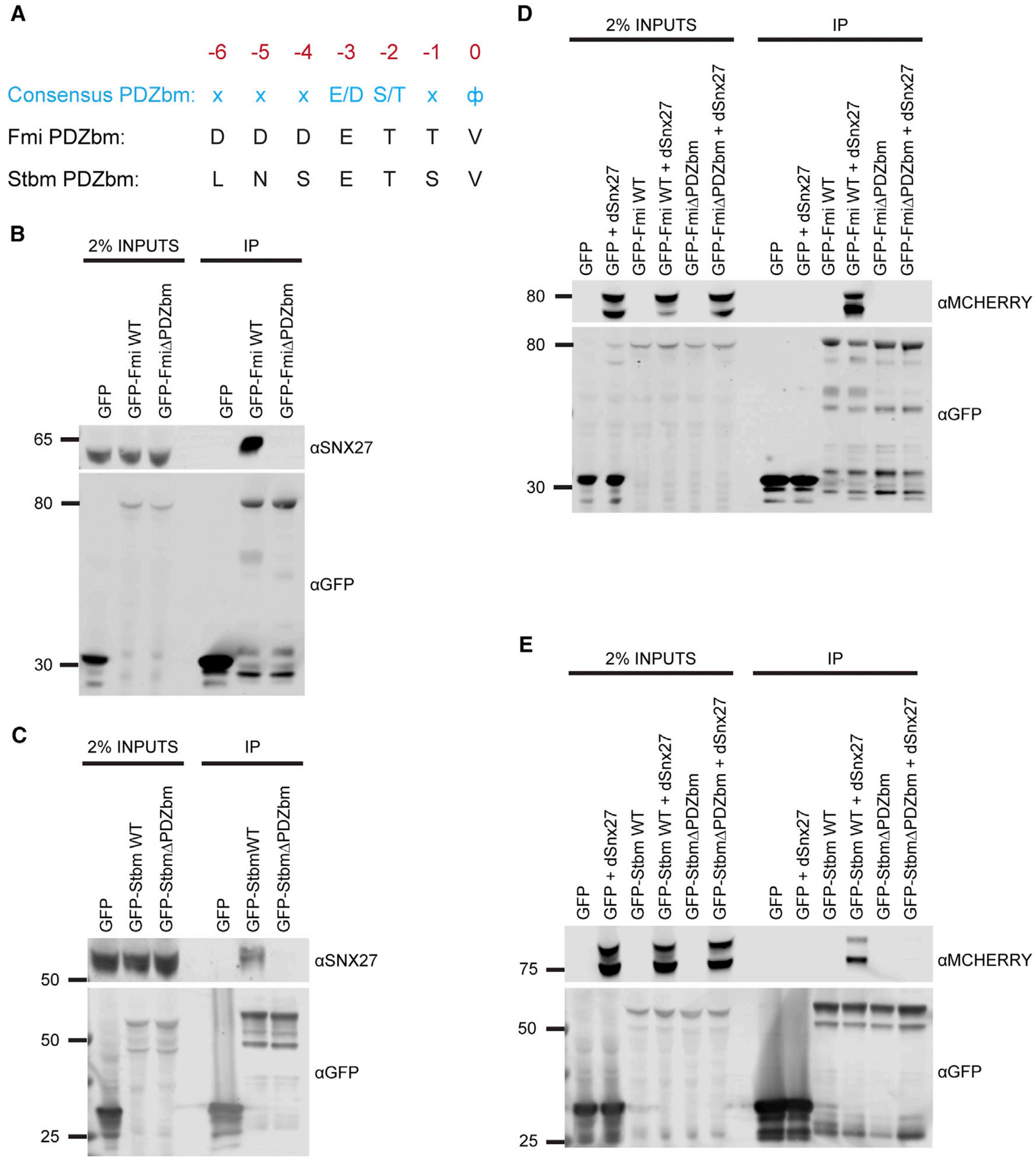

Figure 2. *Drosophila* Snx27 Interacts with the PDZ Binding Motifs Present in the C Termini of Fmi and Stbm

(A) Alignment of the C-terminal PDZ binding motifs of *Drosophila* Fmi and Stbm with the optimized sequence for PDZ binding motif recognition by the PDZ domain of SNX27.

(B–E) GFP-nanotrap immunoprecipitations of HEK293T cells transiently transfected with constructs encoding for GFP-tagged tail regions of Fmi and Stbm (GFP-Fmi wild-type [WT], B and D, and GFP-Stbm WT, C and E) or the corresponding constructs deleted for the last three amino acids of the PDZ binding motif (GFP-FmiΔPDZbm, B and D, and GFP-StbmΔPDZbm, C and E).

(B and C) Samples immunoblotted for the presence of endogenous human SNX27.

(D and E) Cells co-transfected with mCherry-tagged *Drosophila* Snx27 and samples immunoblotted with anti-mCherry. Data are representative of similar data derived from three independent biological replicates.

wings carrying *Snx27* clones were labeled with a pulse of Fmi antibody and then internalization was time resolved after antibody wash off. As expected, extracellular Fmi labeling revealed less junctional Fmi in *Snx27* tissue compared to wild-type (0.64 ± 0.10; $p < 0.001$) at the start of the experiment (Figure 4A). In both wild-type and *Snx27* tissue, extracellular Fmi levels decreased over time (Figures 4A–4C and 4G). This was accompanied by the appearance of Fmi in intracellular puncta, which we previously showed were endosomal (Figures 4D–4F) [23]. Interestingly, at 10 min internalization, a similar number of puncta

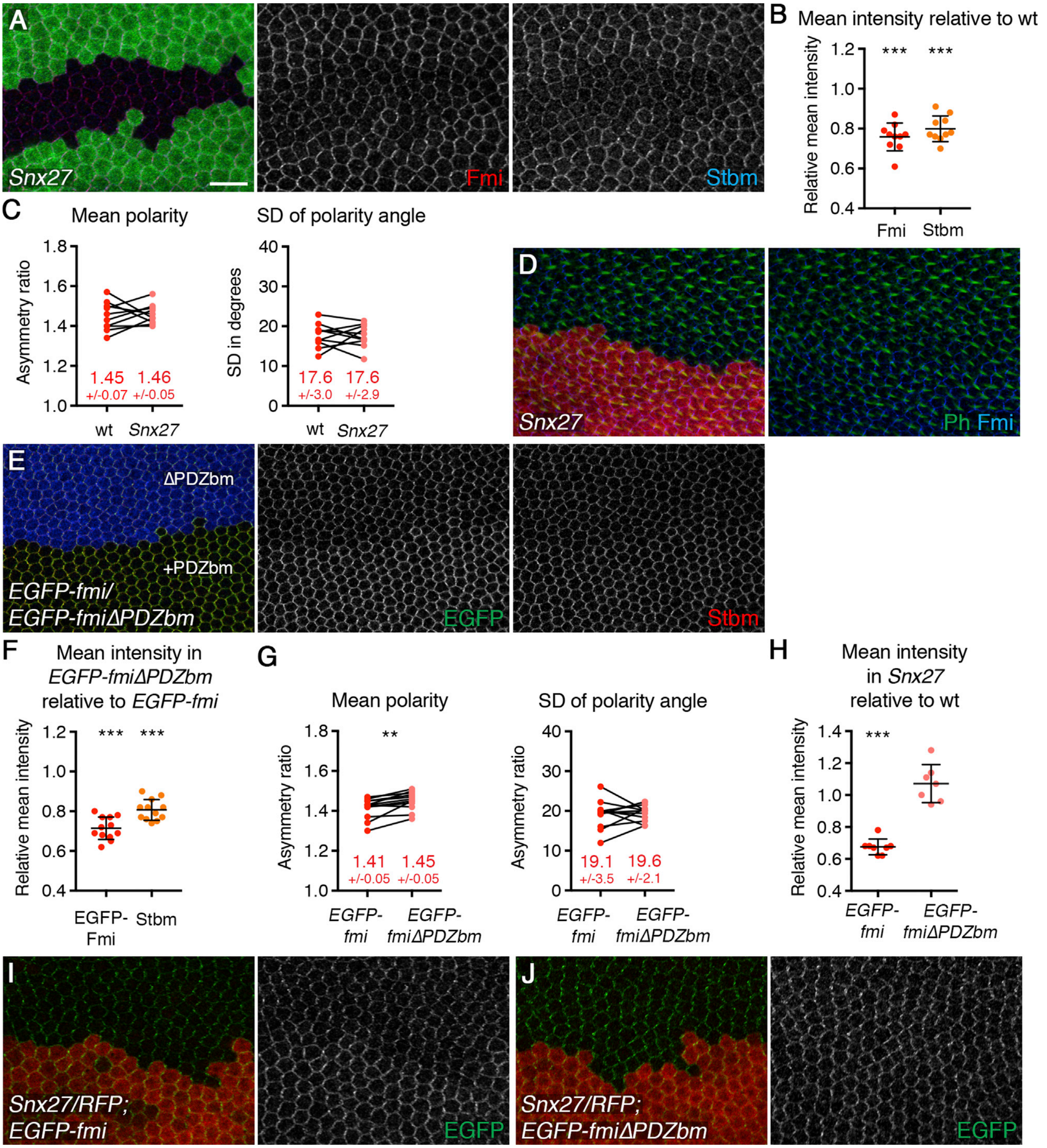

Figure 3. Snx27 Regulates Junctional Levels of Fmi and Stbm via the PDZ Binding Motif of Fmi

(A and D) 28-hr APF (A) or 32-hr APF (D) pupal wings carrying clones of *Snx27* (Figure S2A), marked by loss of GFP immunolabeling (green in A) or RFP fluorescence (red in D). Wings are immunolabeled for Fmi in red and Stbm in blue (A) or Fmi in blue and phalloidin in green (D). Scale bar 10 μm.

(B) Quantitation of mean intensity of Fmi (red dots) or Stbm (orange dots) membrane labeling in pupal wing clones of *Snx27*. Intensity is shown as a ratio of signal in *Snx27* mutant compared to wild-type in each wing.

(C) Mean polarity and variation in polarity angle of wings immunolabeled for Fmi in wild-type and *Snx27* mutant tissue.

(E) 28-hr APF pupal wing with twin clones of *arm-PRO-EGFP-fmi* next to *arm-PRO-EGFP-fmiΔPDZ binding motif* (ΔPDZbm), marked by β-gal immunolabeling in blue, in a fmi^{E59}/fmi^{E45} mutant background. The wing is immunolabeled for EGFP in green and Stbm in red.

(F) Quantitation of mean intensity of membrane labeling of EGFP-Fmi (red dots) and Stbm (orange dots). Intensity is shown as a ratio of signal in ΔPDZbm compared to full-length protein in each wing.

(legend continued on next page)

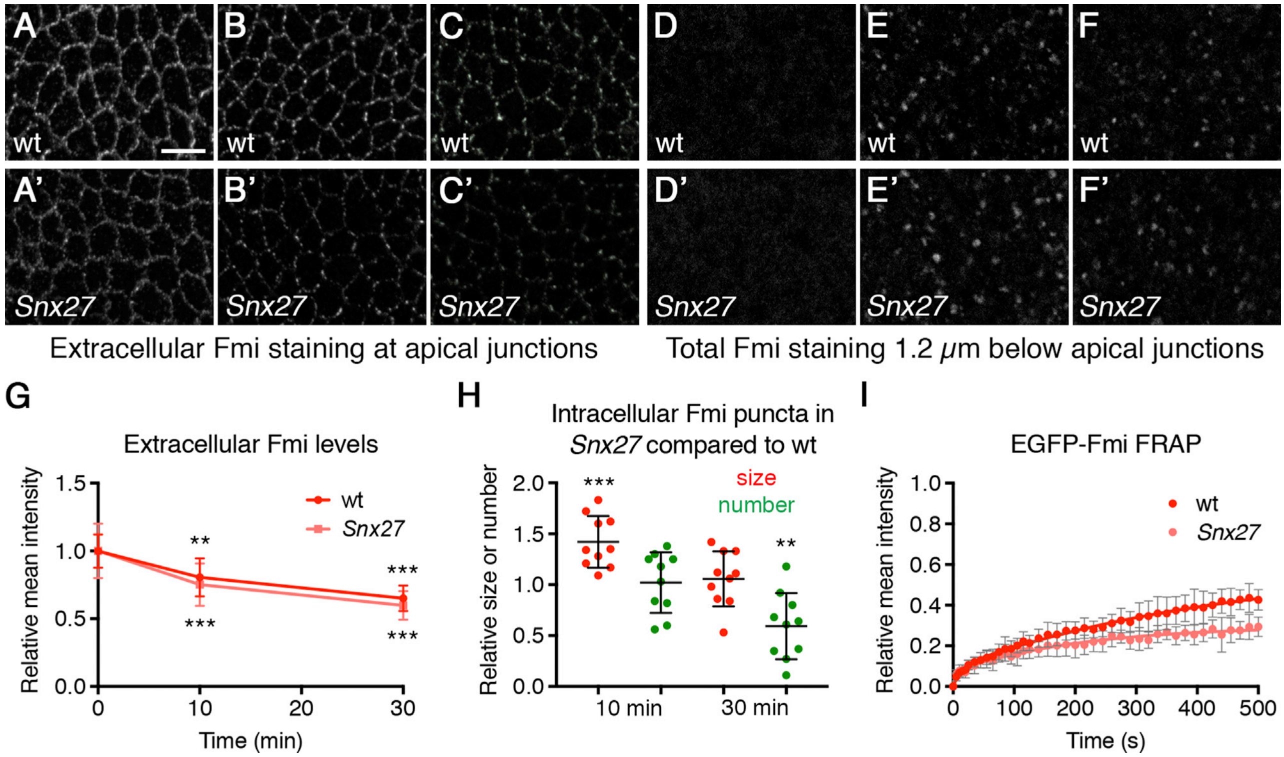

Figure 4. Loss of Snx27 Disrupts Intracellular Trafficking of Fmi

(A–F) Images of Fmi internalization experiment. (A–C) Extracellular Fmi immunolabeling of 5.5 hr APF prepupal wings, imaged at the apical junctions.

(D–F) Total Fmi immunolabeling of the same wings, imaged 1.2 μm below the apical junctions. Images at 0 min (A and D), 10 min (B and E), or 30 min (C and F) after removal of Fmi antibody, in wild-type (A–F) or *Snx27* (A'–F') mutant regions of the same wings are shown. Scale bar 5 μm. (G and H) Quantitation of extracellular Fmi immunolabeling (G) or Fmi intracellular puncta size and number (H) in wild-type and *Snx27* tissue, after antibody internalization.

(G) Intensity at 0 min is normalized to 1.0 for each genotype (but note that Fmi levels in *Snx27* tissue were 64% of levels in wild-type). Error bars are SD; ANOVA with Dunnett's multiple comparisons test was used to compare intensities to 0 min; ***$p < 0.001$; **$p \leq 0.01$. Immunolabeling is more punctate after internalization, as Fmi that is highly clustered is stable, and non-clustered Fmi is rapidly internalized (see [23]).

(H) Data are shown as a ratio of puncta size or number in *Snx27* mutant compared to wild-type in each wing. Error bars are SD. One-sample t tests were used to determine whether the ratio differed from 1.0; ***$p < 0.001$; **$p < 0.01$. Note that puncta intensity was similar in wild-type and *Snx27* tissue.

(I) FRAP of EGFP-Fmi, in 5.5-hr APF prepupal wings from wild-type or *Snx27* flies. Note that pre-bleach levels of EGFP-Fmi in *Snx27* tissue were decreased relative to wild-type (0.56 ± 0.13; $p < 0.001$), but data on the graph were normalized to a pre-bleach intensity of 1.0 for each genotype. A two-phase exponential curve was fitted; error bars are SD. Recovery in the first phase is not significantly different, but there is proportionally less recovery in the second phase in *Snx27* compared to wild-type ($p = 0.006$). Recovery of neither genotype reaches a plateau in the time of imaging.

See Data S1 and Table S1 for all statistical comparisons.

were observed in *Snx27* and wild-type, and puncta were larger in *Snx27* (Figures 4E and 4H). Because less extracellular Fmi labeling was observed in *Snx27* tissue than wild-type (Figure 4A), and internalization rates were similar (Figure 4G), it follows that endosomal puncta are more persistent in *Snx27* than in wild-type. The number of puncta in *Snx27* relative to wild-type was reduced at 30 min internalization, and puncta size was similar (Figures 4F and 4H). This is consistent with a proportion of Fmi being rapidly recycled to the plasma membrane in wild-type, but in *Snx27*, Fmi persists in endosomes and is subsequently degraded.

Fluorescence recovery after photobleaching (FRAP) experiments using *EGFP-fmi* knockin flies provide a further confirmation of altered Fmi dynamics in *Snx27* wings. Recovery of

(G) Mean polarity and variation in polarity angle of wings immunolabeled for EGFP in *EGFP-fmi* and *EGFP-fmiΔPDZbm* tissue.

(H) Quantitation of mean intensity of EGFP-Fmi membrane labeling. Intensity is shown as a ratio of EGFP signal in *Snx27* mutant compared to wild-type in each wing.

(I and J) 28-hr APF pupal wings expressing *Arm-PRO-EGFP-fmi* (I) or *Arm-PRO-EGFP-fmiΔPDZ binding motif* (*ΔPDZbm*) (J) and carrying clones of *Snx27*, marked by loss of RFP (red). EGFP immunolabeling is in green.

(B, F, and H) Error bars are SD. One-sample t tests were used to determine whether the ratio differed from 1.0; ***$p < 0.001$.

(C and G) Values from the same wing are linked by black bars; mean and SD are listed. Paired t tests were used to compare values in the same wing; **$p < 0.01$.

See also Figures S2, S3, and S4 and Data S1 for all statistical comparisons.

EGFP-Fmi fluorescence after bleaching fitted to a two-phase exponential curve, where the fast phase of recovery was similar in wild-type and *Snx27* wings and the slow recovery was reduced in *Snx27* compared to wild-type (Figure 4I; Table S1). These phases likely correspond to recovery by diffusion and endocytosis and recycling, respectively (compare to [24]) and thus suggest reduced recovery via endosomal trafficking in *Snx27* wings.

In the present study, we provide biochemical and genetic evidence to establish that retromer and its associated cargo adaptor Snx27 are important components in the *in vivo* trafficking of the core planar polarity proteins Fmi and Stbm in the *Drosophila* pupal wing. As far as we are aware, this is the first *in vivo* demonstration of a functional coupling between the core planar polarity cargo and an endosomal recycling complex. Interestingly, in humans, planar polarity mutations have been shown to contribute to the etiology of Robinow syndrome, a severe skeletal dysplasia characterized by short limbs and craniofacial anomalies [25–31]. It is intriguing to note that the *SNX27*$^{-/-}$ mouse phenotype is characterized not only by neurological defects but also skeletal dysplasia, including shortened forelimbs and cranial defects [32], which could be attributed to a defect in the robustness of planar polarization.

STAR★METHODS

Detailed methods are provided in the online version of this paper and include the following:

- KEY RESOURCES TABLE
- CONTACT FOR REAGENT AND RESOURCE SHARING
- EXPERIMENTAL MODEL AND SUBJECT DETAILS
  - Genotypes of experimental models
- METHOD DETAILS
  - Cell culture and western blots
  - Generation of CRISPR/Cas9 Snx27 and Fam21 mutants, and EGFP-fmi knockin flies
  - Transgenics
  - Pupal wing immunostainings, imaging, and quantitation
  - Antibody internalisation
  - FRAP
- QUANTIFICATION AND STATISTICAL ANALYSIS
  - Quantification of western blots
  - Quantification of membrane intensity and polarity in pupal wings
  - Quantification of antibody internalisation
  - FRAP processing
- DATA AND SOFTWARE AVAILABILITY

SUPPLEMENTAL INFORMATION

Supplemental Information includes four figures, one table, and two data files and can be found with this article online at https://doi.org/10.1016/j.cub.2018.12.027.

ACKNOWLEDGMENTS

We thank Jessica Gamage for making transgenic constructs; John Walker for making the *EGFP-fmi* knockin; and the Bloomington *Drosophila* Stock Center, the Vienna *Drosophila* Resource Centre, and Avital Rodal for fly stocks. The Developmental Studies Hybridoma Bank is thanked for antibodies and BestGene, Genetivision, and the University of Cambridge Department of Genetics for embryo injections. This work was funded through a Wellcome Senior Fellowship (grant number 100986/Z/13/Z) awarded to D.S. and through Wellcome Trust (104568/Z/14/Z) and the Medical Research Council (MR/L007363/1 and MR/P018807/1) awards to P.J.C. Imaging was performed in the Wolfson Light Microscopy Facility at the University of Sheffield.

AUTHOR CONTRIBUTIONS

Conceptualization, P.J.C. and D.S.; Reagent Generation, Acquisition, and Analysis of Data, H.S., P.F.L., N.P., and K.J.M.; Writing – First Draft, P.J.C., H.S., and N.P., Writing – Review and Editing, H.S., P.F.L., N.P., K.J.M., D.S., and P.J.C.; Funding Acquisition, Resources, and Supervision, D.S. and P.J.C.

DECLARATION OF INTERESTS

The authors declare no competing interests.

Received: August 2, 2018
Revised: November 15, 2018
Accepted: December 14, 2018
Published: January 17, 2019

REFERENCES

1. Butler, M.T., and Wallingford, J.B. (2017). Planar cell polarity in development and disease. Nat. Rev. Mol. Cell Biol. *18*, 375–388.
2. Goodrich, L.V., and Strutt, D. (2011). Principles of planar polarity in animal development. Development *138*, 1877–1892.
3. Devenport, D. (2014). The cell biology of planar cell polarity. J. Cell Biol. *207*, 171–179.
4. Seaman, M.N.J., McCaffery, J.M., and Emr, S.D. (1998). A membrane coat complex essential for endosome-to-Golgi retrograde transport in yeast. J. Cell Biol. *142*, 665–681.
5. Burd, C., and Cullen, P.J. (2014). Retromer: a master conductor of endosome sorting. Cold Spring Harb. Perspect. Biol. *6*, a016774.
6. Wang, S., and Bellen, H.J. (2015). The retromer complex in development and disease. Development *142*, 2392–2396.
7. Cullen, P.J., and Steinberg, F. (2018). To degrade or not to degrade: mechanisms and significance of endocytic recycling. Nat. Rev. Mol. Cell Biol. *19*, 679–696.
8. Franch-Marro, X., Wendler, F., Guidato, S., Griffith, J., Baena-Lopez, A., Itasaki, N., Maurice, M.M., and Vincent, J.P. (2008). Wingless secretion requires endosome-to-Golgi retrieval of Wntless/Evi/Sprinter by the retromer complex. Nat. Cell Biol. *10*, 170–177.
9. Port, F., Kuster, M., Herr, P., Furger, E., Bänziger, C., Hausmann, G., and Basler, K. (2008). Wingless secretion promotes and requires retromer-dependent cycling of Wntless. Nat. Cell Biol. *10*, 178–185.
10. Pocha, S.M., Wassmer, T., Niehage, C., Hoflack, B., and Knust, E. (2011). Retromer controls epithelial cell polarity by trafficking the apical determinant Crumbs. Curr. Biol. *21*, 1111–1117.
11. Zhou, B., Wu, Y., and Lin, X. (2011). Retromer regulates apical-basal polarity through recycling Crumbs. Dev. Biol. *360*, 87–95.
12. de Vreede, G., Schoenfeld, J.D., Windler, S.L., Morrison, H., Lu, H., and Bilder, D. (2014). The Scribble module regulates retromer-dependent endocytic trafficking during epithelial polarization. Development *141*, 2796–2802.
13. Derivery, E., Sousa, C., Gautier, J.J., Lombard, B., Loew, D., and Gautreau, A. (2009). The Arp2/3 activator WASH controls the fission of endosomes through a large multiprotein complex. Dev. Cell *17*, 712–723.
14. Gomez, T.S., and Billadeau, D.D. (2009). A FAM21-containing WASH complex regulates retromer-dependent sorting. Dev. Cell *17*, 699–711.

15. Linardopoulou, E.V., Parghi, S.S., Friedman, C., Osborn, G.E., Parkhurst, S.M., and Trask, B.J. (2007). Human subtelomeric WASH genes encode a new subclass of the WASP family. PLoS Genet. *3*, e237.
16. Dong, B., Kakihara, K., Otani, T., Wada, H., and Hayashi, S. (2013). Rab9 and retromer regulate retrograde trafficking of luminal protein required for epithelial tube length control. Nat. Commun. *4*, 1358.
17. Olivares-Castiñeira, I., and Llimargas, M. (2017). EGFR controls Drosophila tracheal tube elongation by intracellular trafficking regulation. PLoS Genet. *13*, e1006882.
18. Steinberg, F., Gallon, M., Winfield, M., Thomas, E.C., Bell, A.J., Heesom, K.J., Tavaré, J.M., and Cullen, P.J. (2013). A global analysis of SNX27-retromer assembly and cargo specificity reveals a function in glucose and metal ion transport. Nat. Cell Biol. *15*, 461–471.
19. Gallon, M., Clairfeuille, T., Steinberg, F., Mas, C., Ghai, R., Sessions, R.B., Teasdale, R.D., Collins, B.M., and Cullen, P.J. (2014). A unique PDZ domain and arrestin-like fold interaction reveals mechanistic details of endocytic recycling by SNX27-retromer. Proc. Natl. Acad. Sci. USA *111*, E3604–E3613.
20. Clairfeuille, T., Mas, C., Chan, A.S.M., Yang, Z., Tello-Lafoz, M., Chandra, M., Widagdo, J., Kerr, M.C., Paul, B., Mérida, I., et al. (2016). A molecular code for endosomal recycling of phosphorylated cargos by the SNX27-retromer complex. Nat. Struct. Mol. Biol. *23*, 921–932.
21. Bastock, R., Strutt, H., and Strutt, D. (2003). Strabismus is asymmetrically localised and binds to Prickle and Dishevelled during Drosophila planar polarity patterning. Development *130*, 3007–3014.
22. Strutt, H., Gamage, J., and Strutt, D. (2016). Robust asymmetric localization of planar polarity proteins is associated with organization into signalosome-like domains of variable stoichiometry. Cell Rep. *17*, 2660–2671.
23. Strutt, H., Warrington, S.J., and Strutt, D. (2011). Dynamics of core planar polarity protein turnover and stable assembly into discrete membrane subdomains. Dev. Cell *20*, 511–525.
24. Bulgakova, N.A., Grigoriev, I., Yap, A.S., Akhmanova, A., and Brown, N.H. (2013). Dynamic microtubules produce an asymmetric E-cadherin-Bazooka complex to maintain segment boundaries. J. Cell Biol. *201*, 887–901.
25. Afzal, A.R., Rajab, A., Fenske, C.D., Oldridge, M., Elanko, N., Ternes-Pereira, E., Tüysüz, B., Murday, V.A., Patton, M.A., Wilkie, A.O., and Jeffery, S. (2000). Recessive Robinow syndrome, allelic to dominant brachydactyly type B, is caused by mutation of ROR2. Nat. Genet. *25*, 419–422.
26. van Bokhoven, H., Celli, J., Kayserili, H., van Beusekom, E., Balci, S., Brussel, W., Skovby, F., Kerr, B., Percin, E.F., Akarsu, N., and Brunner, H.G. (2000). Mutation of the gene encoding the ROR2 tyrosine kinase causes autosomal recessive Robinow syndrome. Nat. Genet. *25*, 423–426.
27. Person, A.D., Beiraghi, S., Sieben, C.M., Hermanson, S., Neumann, A.N., Robu, M.E., Schleiffarth, J.R., Billington, C.J., Jr., van Bokhoven, H., Hoogeboom, J.M., et al. (2010). WNT5A mutations in patients with autosomal dominant Robinow syndrome. Dev. Dyn. *239*, 327–337.
28. White, J., Mazzeu, J.F., Hoischen, A., Jhangiani, S.N., Gambin, T., Alcino, M.C., Penney, S., Saraiva, J.M., Hove, H., Skovby, F., et al.; Baylor-Hopkins Center for Mendelian Genomics (2015). DVL1 frameshift mutations clustering in the penultimate exon cause autosomal-dominant Robinow syndrome. Am. J. Hum. Genet. *96*, 612–622.
29. Bunn, K.J., Daniel, P., Rösken, H.S., O'Neill, A.C., Cameron-Christie, S.R., Morgan, T., Brunner, H.G., Lai, A., Kunst, H.P., Markie, D.M., and Robertson, S.P. (2015). Mutations in DVL1 cause an osteosclerotic form of Robinow syndrome. Am. J. Hum. Genet. *96*, 623–630.
30. White, J.J., Mazzeu, J.F., Hoischen, A., Bayram, Y., Withers, M., Gezdirici, A., Kimonis, V., Steehouwer, M., Jhangiani, S.N., Muzny, D.M., et al.; Baylor-Hopkins Center for Mendelian Genomics (2016). DVL3 alleles resulting in a −1 frameshift of the last exon mediate autosomal-dominant Robinow syndrome. Am. J. Hum. Genet. *98*, 553–561.
31. White, J.J., Mazzeu, J.F., Coban-Akdemir, Z., Bayram, Y., Bahrambeigi, V., Hoischen, A., van Bon, B.W.M., Gezdirici, A., Gulec, E.Y., Ramond, F., et al.; Baylor-Hopkins Center for Mendelian Genomics (2018). WNT signaling perturbations underlie the genetic heterogeneity of Robinow syndrome. Am. J. Hum. Genet. *102*, 27–43.
32. Chan, A.S., Clairfeuille, T., Landao-Bassonga, E., Kinna, G., Ng, P.Y., Loo, L.S., Cheng, T.S., Zheng, M., Hong, W., Teasdale, R.D., et al. (2016). Sorting nexin 27 couples PTHR trafficking to retromer for signal regulation in osteoblasts during bone growth. Mol. Biol. Cell *27*, 1367–1382.
33. Usui, T., Shima, Y., Shimada, Y., Hirano, S., Burgess, R.W., Schwarz, T.L., Takeichi, M., and Uemura, T. (1999). Flamingo, a seven-pass transmembrane cadherin, regulates planar cell polarity under the control of Frizzled. Cell *98*, 585–595.
34. Strutt, H., and Strutt, D. (2008). Differential stability of flamingo protein complexes underlies the establishment of planar polarity. Curr. Biol. *18*, 1555–1564.
35. Bastock, R., and Strutt, D. (2007). The planar polarity pathway promotes coordinated cell migration during Drosophila oogenesis. Development *134*, 3055–3064.
36. Strutt, H., Searle, E., Thomas-Macarthur, V., Brookfield, R., and Strutt, D. (2013). A Cul-3-BTB ubiquitylation pathway regulates junctional levels and asymmetry of core planar polarity proteins. Development *140*, 1693–1702.
37. Strutt, H., Price, M.A., and Strutt, D. (2006). Planar polarity is positively regulated by casein kinase Iepsilon in Drosophila. Curr. Biol. *16*, 1329–1336.
38. Zhang, P., Wu, Y., Belenkaya, T.Y., and Lin, X. (2011). SNX3 controls Wingless/Wnt secretion through regulating retromer-dependent recycling of Wntless. Cell Res. *21*, 1677–1690.
39. Wang, S., Tan, K.L., Agosto, M.A., Xiong, B., Yamamoto, S., Sandoval, H., Jaiswal, M., Bayat, V., Zhang, K., Charng, W.-L., et al. (2014). The retromer complex is required for rhodopsin recycling and its loss leads to photoreceptor degeneration. PLoS Biol. *12*, e1001847.
40. Wolff, T., and Rubin, G.M. (1998). *Strabismus*, a novel gene that regulates tissue polarity and cell fate decisions in *Drosophila*. Development *125*, 1149–1159.
41. Port, F., Chen, H.M., Lee, T., and Bullock, S.L. (2014). Optimized CRISPR/Cas tools for efficient germline and somatic genome engineering in Drosophila. Proc. Natl. Acad. Sci. USA *111*, E2967–E2976.
42. Baena-Lopez, L.A., Alexandre, C., Mitchell, A., Pasakarnis, L., and Vincent, J.P. (2013). Accelerated homologous recombination and subsequent genome modification in Drosophila. Development *140*, 4818–4825.
43. Huang, J., Zhou, W., Watson, A.M., Jan, Y.N., and Hong, Y. (2008). Efficient ends-out gene targeting in Drosophila. Genetics *180*, 703–707.
44. Carreira-Barbosa, F., Kajita, M., Morel, V., Wada, H., Okamoto, H., Martinez Arias, A., Fujita, Y., Wilson, S.W., and Tada, M. (2009). Flamingo regulates epiboly and convergence/extension movements through cell cohesive and signalling functions during zebrafish gastrulation. Development *136*, 383–392.
45. Strutt, D.I. (2001). Asymmetric localization of frizzled and the establishment of cell polarity in the Drosophila wing. Mol. Cell *7*, 367–375.
46. Aigouy, B., Farhadifar, R., Staple, D.B., Sagner, A., Röper, J.C., Jülicher, F., and Eaton, S. (2010). Cell flow reorients the axis of planar polarity in the wing epithelium of Drosophila. Cell *142*, 773–786.

STAR★METHODS

KEY RESOURCES TABLE

REAGENT or RESOURCE	SOURCE	IDENTIFIER
Antibodies		
mouse monoclonal anti-Fmi #74 (immunolabelling of fixed tissues)	DSHB [33]	RRID: AB_2619583
mouse monoclonal anti-Fmi #71 (antibody internalisation)	[33]	n/a
rat anti-Stbm	[34]	n/a
affinity purified rabbit anti-Fz	[35]	n/a
affinity purified rat anti-Pk	[36]	n/a
rat anti-Dsh	[37]	n/a
mouse monoclonal anti-Arm	DSHB	RRID: AB_528089
mouse monoclonal anti-βgal 40-1a	DSHB	RRID: AB_2314509
rabbit anti β-gal	MP Biochemicals/Cappel	cat# 0855976 (old cat# 55976); RRID: AB_2334934
affinity purified rabbit anti-GFP	Abcam	cat# ab6556; RRID: AB_305564
mouse monoclonal anti-SNX27 1C6	Abcam	cat# AB77799; RRID: AB_10673818
mouse monoclonal anti-GFP	Roche	cat# 11814460001; RRID: AB_390913
mouse monoclonal anti-mCherry	Abcam	cat# AB125096; RRID: AB_11133266
mouse monoclonal anti-Actin AC40	Sigma	cat# A4700; RRID: AB_476730
Alexa Fluor 680 goat anti-mouse IgG	Thermo-Fisher	cat# A21057; RRID: AB_2535723
Alexa Fluor 800 goat anti-rabbit IgG	Thermo-Fisher	cat# SA535571; RRID: AB_2556775
HRP conjugated goat anti Mouse	DAKO	cat# P0447; RRID: AB_2617137
Chemicals, Peptides, and Recombinant Proteins		
Alexa Fluor 568 conjugated Phalloidin	Invitrogen	cat# A12380
FITC conjugated Phalloidin	Molecular Probes	cat# F-432
DMEM	Sigma	cat# D5796
Foetal bovine serum for use with DMEM	Sigma	cat# F7524
GFP trap beads	Chromotek	cat# gta-20
SuperSignal West Dura Extended duration substrate	Thermo-Fisher	cat# 34075
16% paraformaldehyde solution (methanol free)	Agar Scientific	cat# R1026
Triton X-100	VWR	cat# 28817.295; CAS: 9002-93-1
Normal goat serum	Jackson Labs	cat# 005-000-121
Glycerol	VWR	cat# 284546F; CAS: 56-81-5
DABCO	Fluka	cat# 33480; CAS: 280-57-9
Schneider's medium	Thermo-Fisher	cat# 21720
Foetal bovine serum for use with Schneider's medium	Sigma	cat# F4135
Mowiol	Polysciences	cat# 17951; CAS: 9002-89-5
Methyl cellulose	Sigma	cat# 274429; CAS: 9004-67-5
Experimental Models: Cell Lines		
HEK293	ATCC	CRL-11268
Experimental Models: Organisms/Strains		
Vps35^{MH20}	[8]	FlyBase: FBal0221635
Snx1$^{\Delta 2}$	[38]	FlyBase: FBal0336681

(*Continued on next page*)

Continued		
REAGENT or RESOURCE	SOURCE	IDENTIFIER
Snx6^{1}	[38]	FlyBase: FBal0267856
Fam21	This work	n/a
wash^{185}	[15]	FlyBase: FBal0219165
Snx27^{25}	This work	n/a
Vps26^{C}	[39]	FlyBase: FBal0316800
stbm^{6}	[40]	FlyBase: FBal0062423
fmi^{E59}	[33]	FlyBase: FBal0101421
fmi^{E45}	[33]	FlyBase: FBal0101422
tub-GFP-Snx27	This work	n/a
P[acman]-EGFP-stbm	This work	n/a
P[acman]-EGFP-stbmΔPDZ binding motif	This work	n/a
armP-PRO-EGFP-fmi	This work	n/a
armP-PRO-EGFP-fmiΔPDZ binding motif	This work	n/a
EGFP-fmi (knockin allele)	This work	n/a
Ubx-FLP	Bloomington Drosophila Stock Center	FlyBase: FBti0150334
Oligonucleotides		
Snx27 gRNAi 1: GCGAGCCATCCTCACGGC	This work	n/a
Snx27 gRNAi 2: ATCGAAGAGACGCCAATCC	This work	n/a
Fam21 gRNA 1: GCAGGCTTAGGGATGAGCGT	This work	n/a
Fam21 gRNA 1: TTTGATGGAATGGTAGCAGT	This work	n/a
Recombinant DNA		
pEGFP-C1-Fmi	This work	n/a
pEGFP-C1-FmiΔPDZ binding motif	This work	n/a
pEGFP-C1-Stbm	This work	n/a
pEGFP-C1-StbmΔPDZ binding motif	This work	n/a
pmCherry-C1-Snx27	This work	n/a
Software and Algorithms		
Image Studio	LI-COR imaging systems	n/a
Image Lab version 4.1 BioRad Laboratories	BioRad Laboratories	n/a
NIS Elements AR version 4.60	Nikon	n/a
ImageJ version 2.0.0-r65/1.51 s	https://fiji.sc	n/a
Tissue Analyzer	https://grr.gred-clermont.fr/labmirouse/software/WebPA/	n/a
MATLAB_R2014b	Mathworks	n/a
Membrane intensity and Polarity measurement scripts (MATLAB)	[22]	n/a
MATLAB script to compare puncta size	This work	Data S2
GraphPad Prism version 7.0c	GraphPad Software, Inc.	n/a

CONTACT FOR REAGENT AND RESOURCE SHARING

Further information and requests for resources and reagents should be directed to and will be fulfilled by Peter Cullen (Pete.Cullen@bristol.ac.uk).

EXPERIMENTAL MODEL AND SUBJECT DETAILS

HEK293T cells were maintained in DMEM (Sigma, cat# D5796) supplemented with 10% (v/v) fetal bovine serum (Sigma, cat# F7524) under standard conditions.

Drosophila melanogaster flies were grown on standard cornmeal/agar/molasses media at 18°C or 25°C, in plastic vials in a controlled humidity environment, on a 12 hr/12 hr light-dark cycle. For pupal wing dissections, pupae were aged for 28 hr after

puparium formation (APF) at 25°C, or for 32 hr APF for trichome staining. For experiments using prepupal wings, pupae were aged for 5.5 hr APF at 25°C. Sex of pupae was not determined, unless stated below. The health/immune status cannot be determined for individual pupae. Pupae were not subjected to previous procedures and were drug and test naive.

Fly strains are described in the Key Resources Table. *stbm*^{6}, *fmi*^{E59}, *fmi*^{E45}, *Vps35*^{MH20}, *wash*^{185}, *Snx1*$^{\Delta 2}$ and *Snx6*^{1} are putative null mutations. *Vps26*^{C} is a point mutation in a highly conserved residue in the arrestin-like domain. Mitotic clones were generated using the FLP-FRT system and either *Ubx-FLP* or *hs-FLP*.

Genotypes of experimental models

Figure 1

(C-F) *y w Ubx-FLP; FRT42 Vps35*^{MH20} */ FRT42 arm-lacZ*
(D, G) *y w Ubx-FLP; FRT42 Fam21*^{KO} */ FRT42 arm-lacZ*
(D, H) *y w Ubx-FLP; FRT42 wash*^{185} */ FRT42 arm-lacZ*

Figure 3

(A-D) *y w Snx27*^{KO} *FRT19A / y w ubi-GFP FRT19A; Ubx-FLP/+* (females)
(E-G) *y w Ubx-FLP; arm-PRO-EGFP-fmi FRT40 fmi*^{E59} */ arm-PRO-EGFP-fmiΔPDZbm arm-lacZ FRT40 fmi*^{E45}
(H, I) *y w Snx27*^{KO} *FRT19A / Ubi-RFP-nls, w hs-FLP FRT19A; arm-PRO-EGFP-fmi FRT42 fmi*^{E59} / + (females)
(H, J) *y w Snx27*^{KO} *FRT19A / Ubi-RFP-nls, w hs-FLP FRT19A; arm-PRO-EGFP-fmiΔPDZbm FRT42 fmi*^{E59} / + (females)

Figure 4

(A-H) *y w Snx27*^{KO} *FRT19A / Ubi-RFP-nls, w hs-FLP FRT19A; Ubx-FLP* / + (females)
(I) *w*^{1118}*; EGFP-fmi / + (males) and y w Snx27*^{KO} *FRT19A; EGFP-fmi* / + (males)

Figure S1

(A-F) *y w Ubx-FLP; FRT42 Vps35*^{MH20} */ FRT42 arm-lacZ*
(G, I) *y w Ubx-FLP; Snx1*$^{\Delta 2}$ *FRT40 / arm-lacZ FRT40*
(H, I) *y w Ubx-FLP; Snx6*^{1} *FRT40 / arm-lacZ FRT40*

Figure S2

(B, C) *w*^{1118} *(males) and y w Snx27*^{KO} *FRT19A* (males)
(D-H) *y w Snx27*^{KO} *FRT19A / y w ubi-GFP FRT19A; Ubx-FLP/+* (females)
(I, J) *y w Snx27*^{KO} *FRT19A / Ubi-RFP-nls, w hs-FLP FRT19A; tub-GFP-Snx27 / Ubx-FLP* (females)

Figure S3

(A-F) *y w Snx27*^{KO} *FRT19A / Ubi-RFP-nls, w hs-FLP FRT19A; FRT42 Vps35*^{MH20} */ FRT42 arm-lacZ* (females)
(G, H) *y Vps26*^{C} *w FRT19A / Ubi-RFP-nls, w hs-FLP FRT19A; Ubx-FLP* / + (females)

Figure S4

(A, B) *y w Ubx-FLP; P[acman]-EGFP-stbm arm-lacZ FRT40 stbm*^{6} */ P[acman]-EGFP-stbmΔPDZbm FRT40 stbm*^{6}
(C, E) *y w Snx27*^{KO} *FRT19A / Ubi-RFP-nls, w hs-FLP FRT19A; P[acman]-EGFP-stbm FRT40 stbm*^{6} (females)
(D, E) *y w Snx27*^{KO} *FRT19A / Ubi-RFP-nls, w hs-FLP FRT19A; P[acman]-EGFP-stbmΔPDZbm FRT40 stbm*^{6} (females)

METHOD DETAILS

Cell culture and western blots

pEGFP-C1-Stbm and pEGFP-C1-StbmΔPDZbm were generated by amplifying the Stbm C-terminal intracellular tail (encoding the last 285 amino acids for the full-length tail) from genomic DNA (from *w*^{1118} flies) and cloning into the BglII and EcoRI sites of pEGFP-C1 (Clontech). pEGFP-C1-Fmi and pEGFP-C1-FmiΔPDZbm were made by amplifying the C-terminal intracellular tail (encoding the last 527 amino acids for the full-length tail) of Fmi from cDNA and cloning into the XhoI and EcoRI sites of pEGFP-C1. pmCherry-C1-Snx27 was made by amplifying *Snx27-RA* from cDNA and cloning into the NotI and BamHI sites of pmCherry-C1 (Clontech).

Cells were transfected or transduced with the desired GFP-tagged or mCherry-tagged constructs and immunoprecipitations using GFP traps were carried out as previously described [18]. Western blots were performed using standard procedures. Detection was carried out on a Li-Cor Odyssey Infrared scanning system using fluorescently labeled secondary antibodies. Primary antibodies were mouse anti-SNX27 (Clone 1C6, Abcam, cat# AB77799, 1:500), mouse anti-GFP (Roche, cat# 11814460001, 1:1000) and mouse

anti-mCherry (Abcam, cat# AB125096, 1:1000). Secondary antibodies were Alexa Fluor 680 goat anti-mouse IgG (Invitrogen, cat# A21057, 1:10000) and Alexa Fluor 800 goat anti-rabbit IgG (Invitrogen, cat# SA535571, 1:10000). Immunoprecipitations were performed on three independent biological replicates.

For pupal wing westerns, 28 hr APF pupal wings were dissected directly into sample buffer. One pupal wing equivalent was used per lane. Westerns were probed with mouse monoclonal anti-Fmi 74 (DSHB [33],) and Actin AC-40 mouse monoclonal (Sigma, cat# A4700, 1:5000). Secondary antibody was HRP-conjugated goat anti-mouse (DAKO, cat# P0447, 1:5000), and detection used SuperSignal West Dura Extended Duration Substrate (Thermo Scientific, cat# 34075). A BioRad ChemiDoc XRS+ was used for imaging. Western blots were perfomed on four independent biological replicates. No sample size estimation was carried out, and no data were excluded.

Generation of CRISPR/Cas9 Snx27 and Fam21 mutants, and EGFP-fmi knockin flies

CRISPR/Cas9 *Snx27* and *Fam21* mutants were designed using protocols from http://www.crisprflydesign.org and [41], and gRNAs were designed using http://www.flyrnai.org/crispr2/. For *Snx27* the following gRNAs were used: *Snx27* gRNA1 – 5′ GCGAGC CATCCTCACGGC 3′ and *Snx27* gRNA2 – 5′ ATCGAAGAGACGCCAATCC 3′. Guide RNAs were separately cloned into the *pCFD3* vector (http://www.crisprflydesign.org/grna-expression-vectors/ and http://www.addgene.org/Simon_Bullock/) and co-injected into *nos-Cas9* (Bloomington: 54591) embryos by the University of Cambridge Department of Genetics. Progeny were screened by PCR and sequenced, and one mutant allele was obtained, *Snx27*^{25}.

For the *Fam21* knockout, CRISPR/Cas9 was used to generate a 4699 bp deletion, which removes the *Fam21* 5′UTR and all of the coding sequence except the last 36 bp. Gibson assembly was used to insert 1 kb homology arms into the 5′ and 3′ multiple cloning sites of pTV3 (a simplified version of pTV^{cherry} described in [42]. pTV3 sequence available on request) to generate pTV3-Fam21. Two CRISPR targets within proximity of the homology arms, and within the sequence to be deleted, were chosen based on their efficiency and lack of off targets. The 5′ CRISPR target sequence was GCAGGCTTAGGGATGAGCGT (on the plus strand) and the 3′ CRISPR target sequence was TTTGATGGAATGGTAGCAGT (on the minus strand). These protospacer sequences were cloned into the pCFD4 vector [41] to generate pCFD4-Fam21. pTV3-Fam21 and pCFD4-Fam21 were mixed 1:1 and injected at a concentration of 500 ng/ul into *nos-Cas9* (Bloomington: 54591) embryos by Bestgene. The resulting 'founder' adult males were crossed to *y w; G, bc / CyO* and the progeny were screened for mCherry fluorescence, which indicates successful targeting. Knockout alleles were verified by sequencing.

EGFP was knocked into the endogenous *fmi* locus by homologous recombination. The sequence for EGFP was inserted into the pRK2 targeting vector [43], such that EGFP is upstream of the LoxP-white-LoxP cassette. Homology arms of approximately 3 kb of genomic DNA for *fmi* were inserted on either side, inserting EGFP 5 amino acids downstream of the PRO domain and 7 amino acids upstream of the first cadherin repeat, after amino acid Q355.

Targeting vectors were introduced into the genome by P-element mediated transgenesis, to produce donor strains. Homologous recombination was carried out as described by [43]. Targeted lines on the correct chromosome were verified by PCR of EGFP, and the *white* marker gene was excised by Cre-Lox recombination, leaving a single LoxP site downstream of EGFP.

Transgenics

Drosophila Snx27 cDNA (LD13361 Berkeley *Drosophila* Genome Project) was cloned into *pEGFP-C1* (Clontech). *GFP-Snx27* was then cloned into the *tub-MCS* vector and *tub-GFP-Snx27* was integrated into the genome via P-element mediated transgenesis. *P[acman]-EGFP-stbm* was made by inserting an in-frame EGFP tag upstream of the *stbm* ORF in *P[acman]-stbm*, using standard recombineering methods, leaving a LoxP site between EGFP and the *stbm* ORF. For *P[acman]-EGFP-stbmΔPDZbm*, the final three amino acids of the *stbm* ORF were precisely deleted using recombineering with positive-negative selection. *P[acman]-EGFP-stbm* and *P[acman]-EGFP-stbmΔPDZbm* were integrated into the genome via ΦC31-mediated recombination into the *attP40* landing site and recombined with *arm-lacZ FRT40 stbm*^{6} or *FRT40 stbm*^{6} respectively.

To make *arm-PRO-EGFP-fmi*, cDNA from the *stan-PA* isoform was used, which contains a PDZbm at its C terminus. PCR was used to insert EGFP in frame after Q355 in the N terminus of Fmi, 5 amino acids downstream of the predicted PRO cleavage site [44], and 7 amino acids upstream of the start of the first cadherin repeat. The ORF was then inserted downstream of the *armadillo* promoter and upstream of a polyA sequence in a modified *pAttB* vector. PCR was used to delete the final three amino acids to make *arm-PRO-EGFP-fmiΔPDZbm*. *arm-PRO-EGFP-fmi* and *arm-PRO-EGFP-fmiPDZbm* were integrated into the genome via ΦC31-mediated recombination into the *attP40* landing site and recombined with *FRT40 fmi*^{E59} or *arm-lacZ FRT40 fmi*^{E45} respectively.

Transgenics were made by Bestgene or Genetivision.

Pupal wing immunostainings, imaging, and quantitation

Pupal wings were dissected at 28 hr after puparium formation (APF) at 25°C, or at 32 hr APF for trichomes [45]. Pupae were removed from their pupal case and fixed for 25-60 min in 4% paraformaldehyde in PBS, depending on antibody combinations. Wings were then dissected and the outer cuticle removed, and were blocked for 1 hr in PBS containing 0.2% Triton X-100 (PTX) and 10% normal goat serum. Primary and secondary antibodies were incubated overnight at 4°C in PTX with 10% normal goat serum, and all washes were in PTX. After immunolabelling, wings were post-fixed in 4% paraformaldehyde in PBS for 30 min. Wings were mounted in 25 μl of PBS containing 10% glycerol and 2.5% DABCO, pH7.5. Antibodies used for immunolabelling were mouse monoclonal anti-Fmi 74 (DSHB [33],), rat anti-Stbm [34], affinity-purified rabbit anti-Fz [35], affinity-purified rat anti-Pk [36], rat anti-Dsh [37], mouse

monoclonal anti-Armadillo (DSHB), affinity-purified rabbit anti-GFP (Abcam, cat# ab6556), rabbit anti-β-galactosidase (MP Biomedicals/Cappel, cat# 55976) and mouse monoclonal anti-β-galactosidase 40-1a (DSHB). Trichomes were stained using fluorescein Phalloidin (Molecular Probes, cat# F-432) or Alexa Fluor 568 Phalloidin (Invitrogen cat# A12380). Pupal wings were imaged on a Nikon A1R GaAsP confocal microscope using a 60x NA1.4 apochromatic lens. Nine Z-slices separated by 150 nm were imaged. Wings from at least five independent animals were imaged for each experiment. No randomization, blinding or sample size estimation was carried out, and no data were excluded.

Antibody internalisation

5.5 hr APF prepupal wings were dissected in Schneider's Medium (SM; Thermo-Fisher, cat# 21720) containing 10% fetal bovine serum (FBS; Sigma, cat# F4135) and transferred to a microtiter plate on ice. Medium was replaced with SM/FBS containing mouse monoclonal Fmi antibody 71 [33], and wings were incubated at 4°C for 30 min. Wings were washed briefly in SM/FBS on ice, and chased in 1 mL SM/FBS at room temperature (RT) for various times. Endocytosis was stopped by pipetting wings into SM/FBS at 4°C for 5 min, and wings were then fixed in 4% paraformaldehyde/PBS for 20 min. For detection of extracellular Fmi, tissue was incubated in Cy2-conjugated anti-mouse secondary antibody in the absence of detergent, and post-fixed before adding Alexa 647-conjugated anti-mouse secondary antibody in the presence of 0.1% Triton X-100, for total Fmi staining. Wings were mounted in Mowiol (Polysciences, cat# 17951) containing 2.5% DABCO. Pupal wings were imaged on a Nikon A1R GaAsP confocal microscope using a 60x NA1.4 apochromatic lens. Nineteen Z-slices separated by 150 nm were imaged using constant confocal settings. Wings from ten independent animals were imaged. No randomization, blinding or sample size estimation was carried out, and no data were excluded

FRAP

For FRAP, 5.5 hr APF prepupal wing were mounted in a small volume of SM containing 1.25% methyl cellulose (Sigma cat# 274429) to reduce sample movement, and imaged within 45 min. Images were 256 × 256 pixels, with a pixel size of 100 nm, and a pinhole of 1.2 AU. 'Hub-and-spoke' ROIs of 3-4 μm^2 were selected, that covered a vertex and 3 half-cell edges. Three pre-bleach images were taken at 2 frames/sec, and ROIs were then bleached using a 488 nm Argon laser at 80% with 8 passes (1 s total time), which resulted in 60%–75% bleaching. Immediately following bleaching, 5 images were taken at 5 s intervals, followed by 10 images at 10 s intervals and 26 images at 15 s intervals. Laser power was adjusted to maintain constant power between different imaging sessions. Wings from ten independent animals were imaged. Based on the mean intensity and standard deviation of previous sets of wings, we determined that imaging at least 6 wings per genotype would allow detection of differences of 20% in the means, in a pairwise comparison, with a power of 0.8 and α 0.05 (using G*Power). No randomization or blinding was carried out. The last five or six time points from two images were excluded, as the image moved out of focus during imaging.

QUANTIFICATION AND STATISTICAL ANALYSIS

Where the sample size was large enough, a D'Agostino and Pearson normality test was used to determine if the data fitted to a normal distribution. All datasets analyzed had a normal distribution, so parametric statistical tests were performed.

Quantification of western blots

Band intensities from four biological replicates were quantified using ImageJ. Data were compared using unpaired t tests.

Quantification of membrane intensity and polarity in pupal wings

The three brightest slices around apicolateral junctions were selected and averaged for each channel in ImageJ. Membrane masks were generated in Packing Analyzer [46], and MATLAB scripts were used to calculate mean membrane intensity for mutant or wild-type regions of each wing [22]. Polarity magnitude (maximum asymmetry ratio on a cell-by-cell basis) and variation in polarity angle were also calculated using MATLAB [22]. Error bars represent standard deviation. Values were compared between control and mutant regions of the same wings using paired t tests. Alternatively, one sample t tests were used to determine if the ratio of signal between mutant and wild-type regions of the same wing differed from 1.0 ($p \leq 0.05$ *, $p \leq 0.01$ **, $p \leq 0.001$ ***).

Quantification of antibody internalisation

For quantification of extracellular staining, ImageJ was used to select and average the three brightest slices around apicolateral junctions, and to measure total signal in mutant or wild-type regions of each wing. Laser-off background was subtracted, and the readings were normalized to 1.0 at the 0 min time point for each genotype. Error bars represent standard deviation, and ANOVA with Dunnett's multiple comparisons test was used to compare intensities to the 0 min control ($p \leq 0.05$ *, $p \leq 0.01$ **, $p \leq 0.001$ ***).

Intracellular puncta were quantified from three slices just below the level at which junctional staining was no longer visible. A modified version of our MATLAB script [22] was used to find a threshold value that resulted in 1% of the total area in wild-type wings being identified as puncta. The same threshold value was then applied to mutant tissue of the same wing. The number, size, and intensity of puncta was determined, and error bars represent standard deviations. One sample t tests were used to determine if the ratio of values between mutant and wild-type regions of the same wing differed from 1.0 ($p \leq 0.05$ *, $p \leq 0.01$ **, $p \leq 0.001$ ***).

FRAP processing

ImageJ was used to manually reselect up to six bleached regions in each image for each time point. The laser off background was subtracted, and the values were corrected for acquisition bleaching and normalized against the average of the prebleach values. Data were then plotted on an xy graph using Prism (v7 Graphpad) and bleached regions within the same wing were averaged. A two-phase exponential curve was fitted for each wing, as this was preferred over a one-phase exponential fit in Prism. Ten wings ($n = 10$) were then combined per genotype and an exponential association curve was fitted. Curves were compared using an extra-sum-of-squares F test ($p \leq 0.05$ *, $p \leq 0.01$ **, $p \leq 0.001$ ***).

DATA AND SOFTWARE AVAILABILITY

A MATLAB script for comparing puncta intensities in two regions of the same wing, using the same threshold value to identify puncta, is supplied as Data S2.

Current Biology, Volume *29*

Supplemental Information

Retromer Controls Planar Polarity Protein Levels

and Asymmetric Localization

at Intercellular Junctions

Helen Strutt, Paul F. Langton, Neil Pearson, Kirsty J. McMillan, David Strutt, and Peter J. Cullen

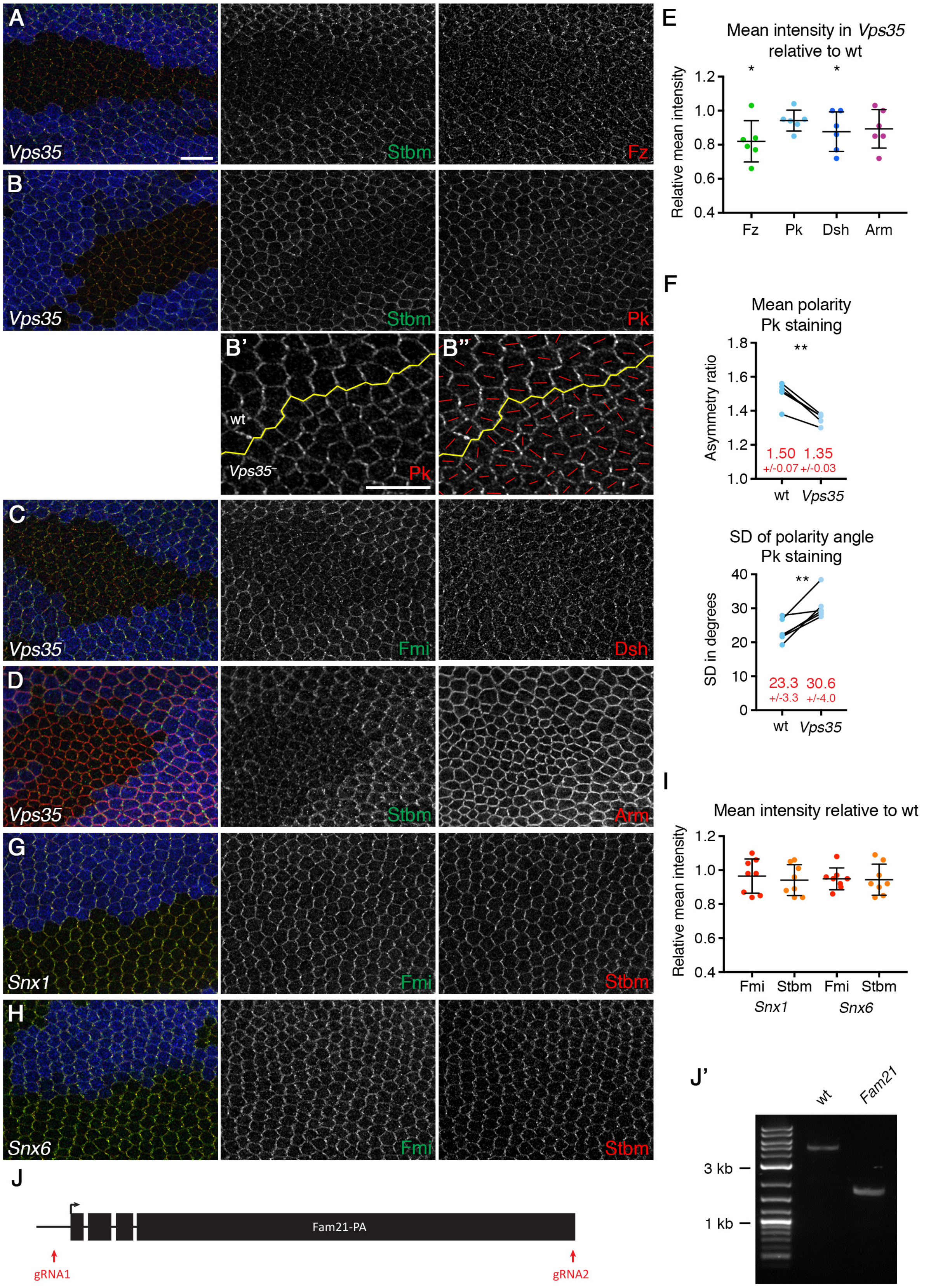

Figure S1. Effects of loss of Vps35, Snx1 and Snx6 on membrane-associated proteins. Related to Figure 1

(A-D, G, H) 28 hr APF pupal wings carrying clones of *Vps35* (A-D), *Snx1* (G) or *Snx6* (H), marked by loss of ß-gal immunolabelling (blue). (A, B, D) Wings immunolabelled for Stbm in green and Fz (A), Pk (B) or Arm (D) in red. (C) Wings immunolabelled for Fmi in green and Dsh in red. (G, H) WIngs immunolabelled for Fmi in

green and Stbm in red. (B') High magnification image of wild-type and mutant regions immunolabelled with Pk and used to quantitate polarity. (B'') Polarity nematic showing the magnitude and angle of polarisation for each cell. Scale bar 10 µm.

(E, I) Quantitation of mean intensity of membrane labelling for the genotypes in A-D, G, H. (E) Wings immunolabelled for Fz (green dots), Pk (pale blue dots), Dsh (dark blue dots) or the non planar polarity protein Armadillo (purple dots). (I) Wings immunolabelled for Fmi (red dots) and Stbm (orange dots). Intensity is shown as a ratio of signal in mutant tissue compared to wild-type in each wing, error bars are SD. One-sample t-tests were used to determine if the ratio differed from 1.0. *$p<0.05$.

(F) Quantitation of polarity in *Vps35* pupal wing clones immunolabelled for Pk, levels of which are not significantly decreased within the clone (see panels B and E). Graphs show mean polarity and variation in polarity angle, in wild-type and *Vps35* mutant tissue. Values from the same wing are linked by black bars, mean and SD are listed. Paired t-tests were used to compare values in the same wing, **$p<0.01$. Like wings immunolabelled for Fmi (Fig. 1E), mean polarity and variation in polarity angle are significantly different in *Vps35* tissue than in wild-type tissue, suggesting that the polarity measurements are not affected by overall levels of protein at the junctions.

(J) Diagram of the *Fam21* locus, showing the exon structure and the position of the gRNAs used for CRISPR/Cas9 gene editing, which results in a replacement of the entire coding sequence of *Fam21*, except the final 36bp, by a pax-mCherry selection cassette. (J') Agarose gel showing PCR products after amplification of genomic DNA from wild-type and *Fam21* homozygous mutant flies. The PCR product in *Fam21* flies is smaller than in wild-type flies, as expected.

See Data S1 for all statistical comparisons.

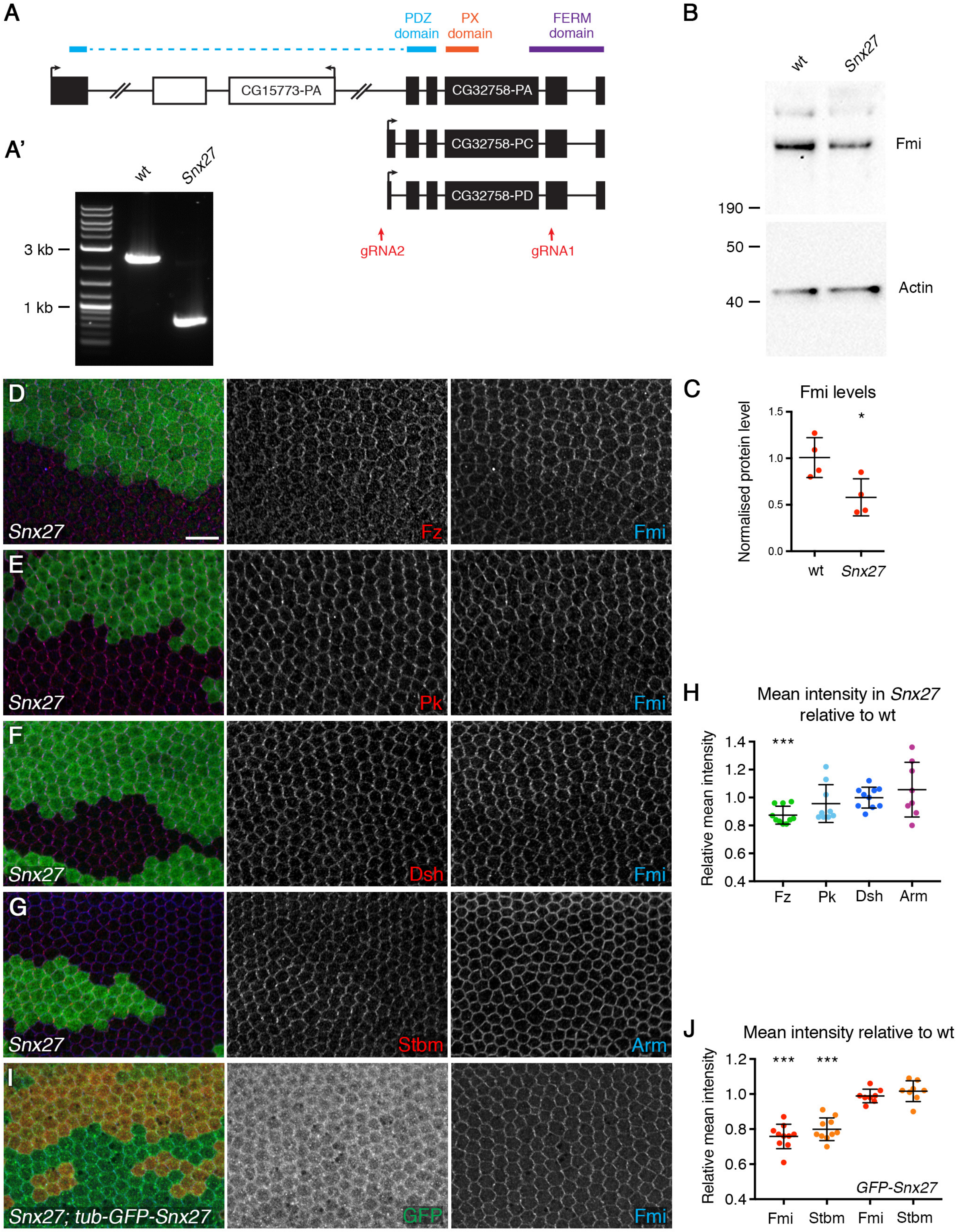

Figure S2. Effect of loss of Snx27 on overall Fmi levels and on other membrane-associated proteins. Related to Figure 3

(A) Diagram of the *Snx27/CG32758* locus showing the 3 predicted isoforms. The sequence encoding the PDZ, PX and FERM domains is indicated above. Another gene (*CG15773*) is present in the first intron of *CG32758-RA*, so the *Snx27* knock-out was made by deleting the C-terminal exons, using two gRNAs targeting the indicated locations. (A') Agarose gel showing PCR products after amplification of genomic DNA from wild-

type and *Snx27* homozygous mutant flies. The PCR product in *Snx27* flies is smaller than in wild-type flies, as expected.

(B-C) Immunoblot (B) and quantitation of protein levels (C) from pupal wing extracts from wild-type and *Snx27* flies, probed for Fmi and Actin. Quantitation was from 4 biological replicates, and samples were compared by unpaired t-test, *p<0.05.

(D-G) 28 hr APF pupal wings carrying clones of *Snx27*, marked by loss of GFP (green). (D-F) Wings immunolabelled for Fmi in blue and Fz (A), Pk (B) or Dsh (C) in red. (G) Wings immunolabelled for Stbm in red and the non-planar polarity protein Armadillo in blue. Scale bar 10 µm.

(I) 28 hr APF pupal wing expressing *tub-GFP-Snx27* (GFP immunolabelling in green) and carrying clones of *Snx27*, marked by loss of RFP fluorescence (red). Wings immunolabelled for Fmi in blue.

(H, J) Quantitation of mean intensity of membrane labelling for the genotypes in D-G, I and Figure 3A. (H) Wings immunolabelled for Fz (green dots), Pk (pale blue dots), Dsh (dark blue dots) or Armadillo (purple dots). (J) Wings immunolabelled for Fmi (red dots) or Stbm (orange dots), in the presence or absence of *tub-GFP-Snx27.* Intensity is shown as a ratio of signal in *Snx27* mutant compared to wild-type in each wing, error bars are SD. One-sample t-tests were used to determine if the ratio differed from 1.0. ***p<0.001.

See Data S1 for all statistical comparisons.

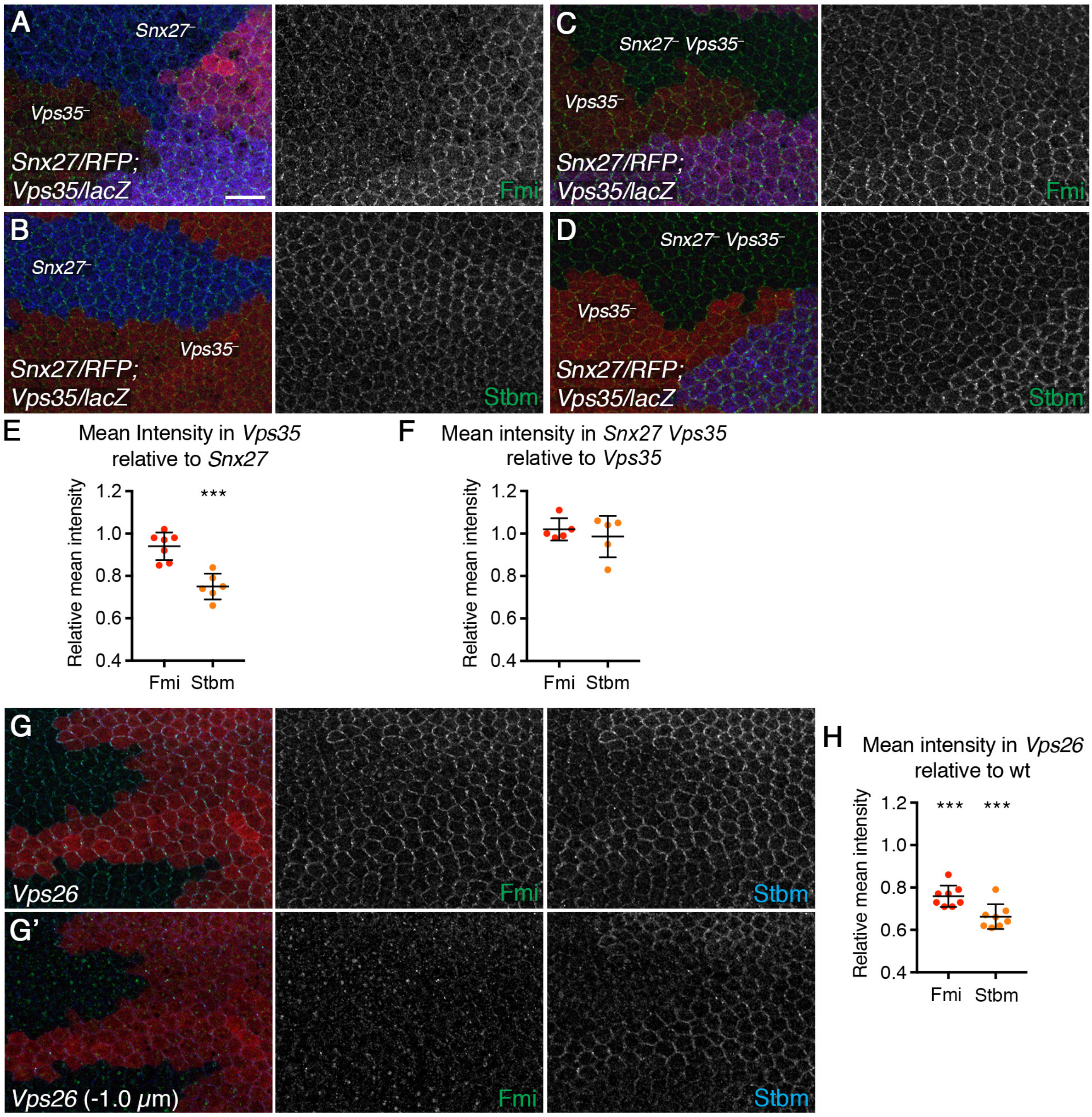

Figure S3. Stbm levels are regulated by retromer independently of Snx27. Related to Figure 3

(A, B, C, D) 28 hr APF pupal wings carrying clones of *Snx27* marked by loss of RFP fluorescence (red), and clones of *Vps35*^{MH20} marked by loss of ß-gal immunolabelling (blue). Wings immunolabelled for Fmi (A, C) or Stbm (B, D) in green. (A, B) *Snx27* clones adjacent to *Vps35* clones. Stbm levels are reduced in *Vps35* clones compared to *Snx27* clones, while Fmi levels are similar. (C, D) Clones of *Vps35* (loss of blue labelling) next to tissue mutant for both *Snx27* and *Vps35* (loss of red and blue labelling). Levels of Fmi and Stbm are similar in both regions. Scale bar 10 µm.

(E, F) Quantitation of mean intensity of Fmi (red dots) or Stbm (orange dots) immunolabelling at membranes for the immunolabellings shown in A, B, C and D. Intensity is shown as a ratio of signal in *Vps35* mutant compared to *Snx27* mutant in each wing (E), or as ratio of signal in *Snx27 Vps35* double mutant compares to *Vps35* single mutant (F). Error bars are SD, one-sample t-tests were used to determine if the ratio differed from 1.0, ***$p<0.001$.

(G) 28 hr APF pupal wings carrying clones of *Vps26*, marked by loss of RFP fluorescence (red). WIngs immunolabelled for Fmi in green and Stbm in blue. (G) shows immunolabelling in the junctional region and (G') shows immunolabelling more basally. Intracellular puncta containing Fmi, but not Stbm, are visible in *Vps26* mutant tissue. Such large intracellular puncta are never seen in *Snx27* mutant tissue, possibly because Fmi never engages with the recycling machinery in this situation and is rapidly degraded in the lysosome. Intracellular

puncta are also rarely seen in *Vps35* mutant tissue. We hypothesise that in *Vps26* mutant tissue, Fmi binds to its cargo adaptor Snx27 and is retained in the endosomal system.

(H) Quantitation of mean intensity of Fmi (red dots) or Stbm (orange dots) membrane labelling in pupal wing clones of *Vps26*. Intensity is shown as a ratio of signal in *Vps26* mutant compared to wild-type in each wing. Error bars are SD, one-sample t-tests were used to determine if the ratio differed from 1.0, ***$p<0.001$.

See Data S1 for all statistical comparisons.

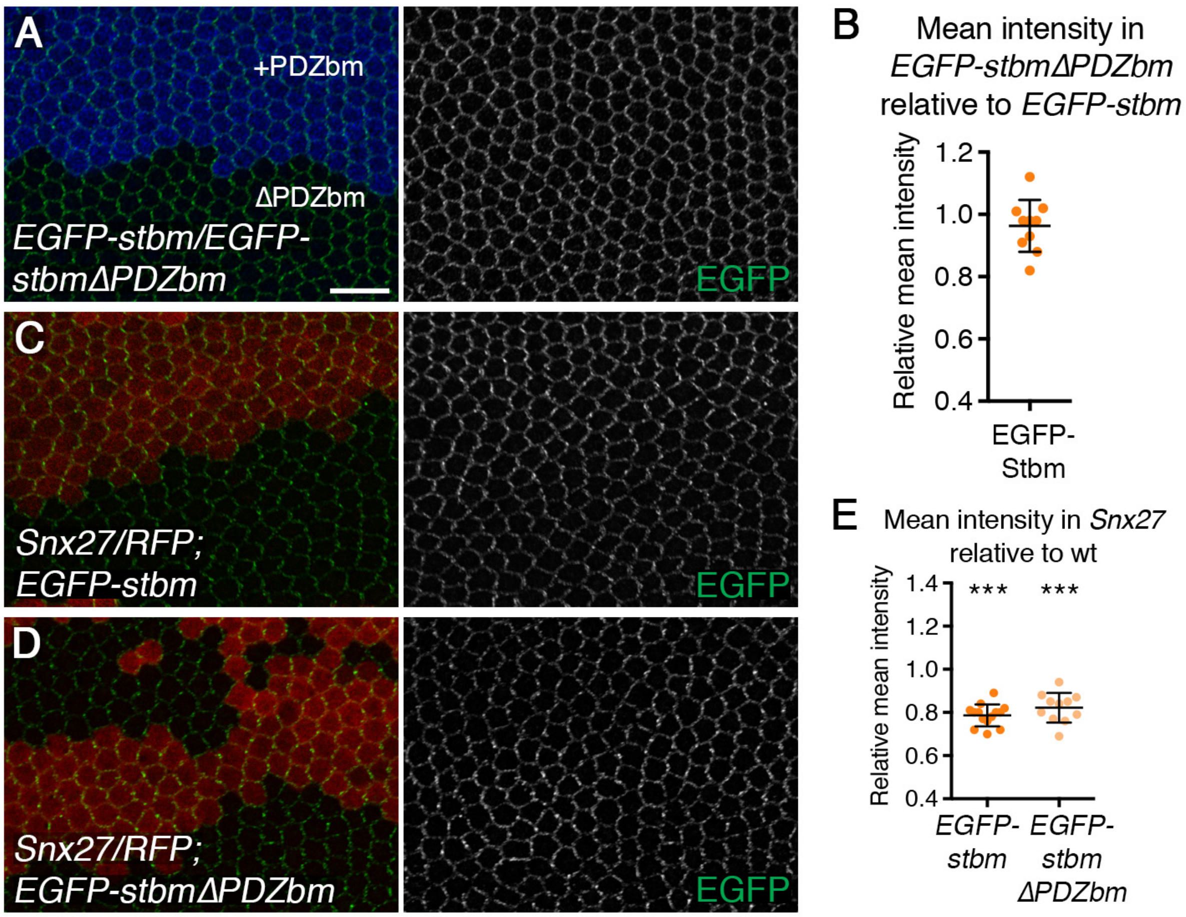

Figure S4. Stbm lacking its PDZ binding motif is sensitive to loss of Snx27. Related to Figure 3

(A) 28 hr APF pupal wings with twin clones of *P[acman]-EGFP-stbm*, marked by ß-gal immunolabelling in blue, next to *P[acman]-EGFP-stbmΔPDZ binding motif* (ΔPDZbm), in a *stbm*^{6} mutant background. Images immunolabelled for EGFP in green. Scale bar 10 µm.

(B) Quantitation of mean intensity of membrane labelling of EGFP-Stbm (A). Intensity is shown as a ratio of signal in ΔPDZbm compared to full-length protein in each wing, error bars are SD. One-sample t-tests were used to determine if the ratio differed from 1.0, no significant differences were seen.

(C, D) 28 hr APF pupal wings expressing *P[acman]-EGFP-stbm* (C) or *P[acman]-EGFP-stbm ΔPDZ binding motif (ΔPDZbm)* (D), in a *stbm*^{6} mutant background. Clones of *Snx27* are marked by loss of RFP (red) and EGFP fluorescence is in green. Levels of both EGFP-Stbm and EGFP-StbmΔPDZbm decrease in *Snx27* clones.

(E) Quantitation of mean intensity of EGFP-Stbm at membranes for the genotypes in C and D. Intensity is shown as a ratio of EGFP signal in *Snx27* mutant compared to wild-type in each wing. Error bars are SD. One-sample t-tests were used to determine if the ratio differed from 1.0, ***$p<0.001$.

See Data S1 for all statistical comparisons.

	wild-type	*EGFP-fmi*	Significance
**Plateau**	**0.60**<br>0.50 - 1.22	**0.30**<br>0.28 - 0.37	**0.006**
**Fast half-life**	**9.2 s**<br>2.4 - 21.8 s	**2.7 s**<br>0.0 - 11.2 s	**0.23**
**Slow half-life**	**313 s**<br>204 - 1029 s	**144 s**<br>107 - 292 s	**0.07**
**Percent Fast recovery**	**15.2**<br>11.9 - 18.5	**24.1**<br>18.9 - 32.2	**different**<br>(model for Percent Fast the same cannot be fitted}

Table S1. Summary of FRAP data. Related to Figure 4

Recovery was fitted to a two-phase exponential curve. Plateau values, fast and slow half lives and the percentage recovery due to the fast phase are shown, together with 95% confidence intervals. Curves were compared using an extra-sum-of squares F test. Note that the curves do not reach a plateau in the time course of the experiment, so the plateaux and half lives are extrapolated, leading to wide 95% confidence intervals. Related to Figure 4I.
